# Supplementary material for: E3 Ligase RNF126 Directly Ubiquitinates Frataxin, Promoting Its Degradation: Identification of a Potential Therapeutic Target for Friedreich Ataxia
Source: Cell Rep. 2017 Feb 21;18(8):2007–17. doi: 10.1016/j.celrep.2017.01.079 (PMC5329121; doi:10.1016/j.celrep.2017.01.079)
Supplement: Document S1. Figures S1–S6 and Tables S1 and S2 [file mmc1.pdf]

Cell Reports, Volume *18*

Supplemental Information

E3 Ligase RNF126 Directly Ubiquitinates Frataxin, Promoting Its Degradation: Identification of a Potential Therapeutic Target for Friedreich Ataxia

Monica Benini, Silvia Fortuni, Ivano Condò, Giulia Alfedi, Florence Malisan, Nicola Toschi, Dario Serio, Damiano Sergio Massaro, Gaetano Arcuri, Roberto Testi, and Alessandra Rufini

SUPPLEMENTAL INFORMATION

Supplemental Figure 1 – Related to Figure 1

A

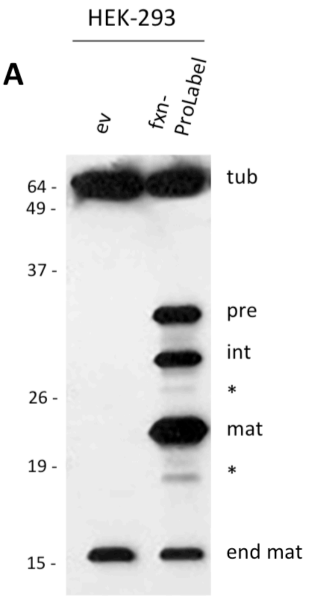

B

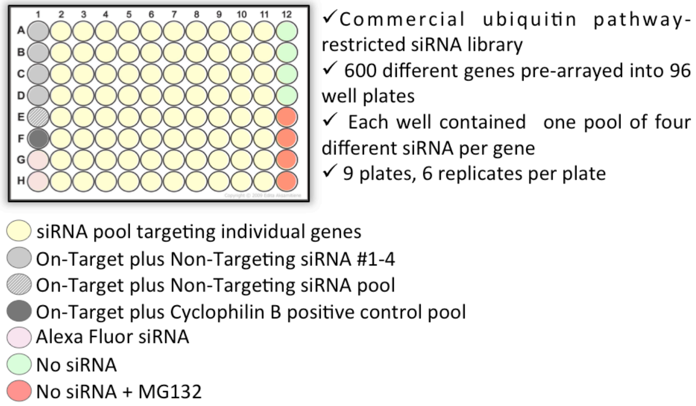

C

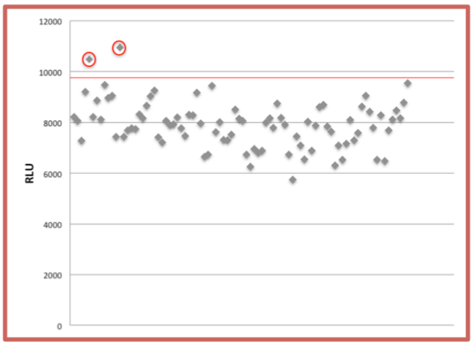

D

Positive replicates (out of 6)	Hit name	Median of NMD across replicates	Median deviation of NMD across replicates	Average FI across replicates	S.D. of FI across replicates
6	Cand1	7.54042	0.660279	1.61	0.16
6	RNF126	6.02534	0.330208	1.49	0.15
6	FBXO44	5.67972	0.549082	1.51	0.15
5	Syvn1	4.78579	1.03014	1.49	0.31
4	FBXW12	5.63934	0.993848	1.35	0.16
4	Cul1	3.87267	0.618967	1.29	0.12

Figure S1. Details of the screening set-up and hits identification.

A) Regular processing of the human frataxin precursor in the ProLabel fusion construct. HEK-293 cells were transiently transfected with empty vector (ev) or frataxin$^{1\text{-}210}$-ProLabel fusion construct (fxn-ProLabel) and analyzed by WB with anti-frataxin and anti-tubulin antibody. Pre: precursor; int: intermediate; mat: mature

frataxin forms expressed by the fxn-ProLabel construct; end mat: endogenous mature. The asterisks (*) denote non-specific bands.

B) Plate layout of Human ON-TARGETplus ubiquitin conjugation siRNA library (Dharmacon). Validated individual control siRNAs and pools are pre-dispensed into column 1 of each RTF library plate, providing a consistent baseline for screening and assay efficiency. Cells were transfected with Alexa Fluor siRNA in the indicated wells to control transfection efficiency. A column of no-siRNA transfected cells was included in each plate and cells treated with the proteasome inhibitor MG132 were included as positive control (column 12).

C) Representative results obtained in one of the screening plate. The graph shows the results obtained from the luminometer reading of one of the plates used for the screening. Each dot represents the RLU (Relative Luminescence Unit) measured in each individual well, corresponding to a determined siRNA pool. The red line indicates the arbitrary threshold that was set as the median of the luminescence measured in all the wells of the plate plus three median absolute deviations. Red dots highlight siRNA pools that were considered positive in this plate. Six replicates per plate were analyzed. siRNA pools that scored above the threshold in at least 4 out of 6 replicates were selected for further validation.

D) Table illustrating the primary hits identified from the siRNA screening. NMD: number of median deviations above the plate median (NMD > 3 was considered a hit on any single replicate). FI: fold increase over plate median (before normalization).

Supplemental Figure 2 – Related to Figure 2

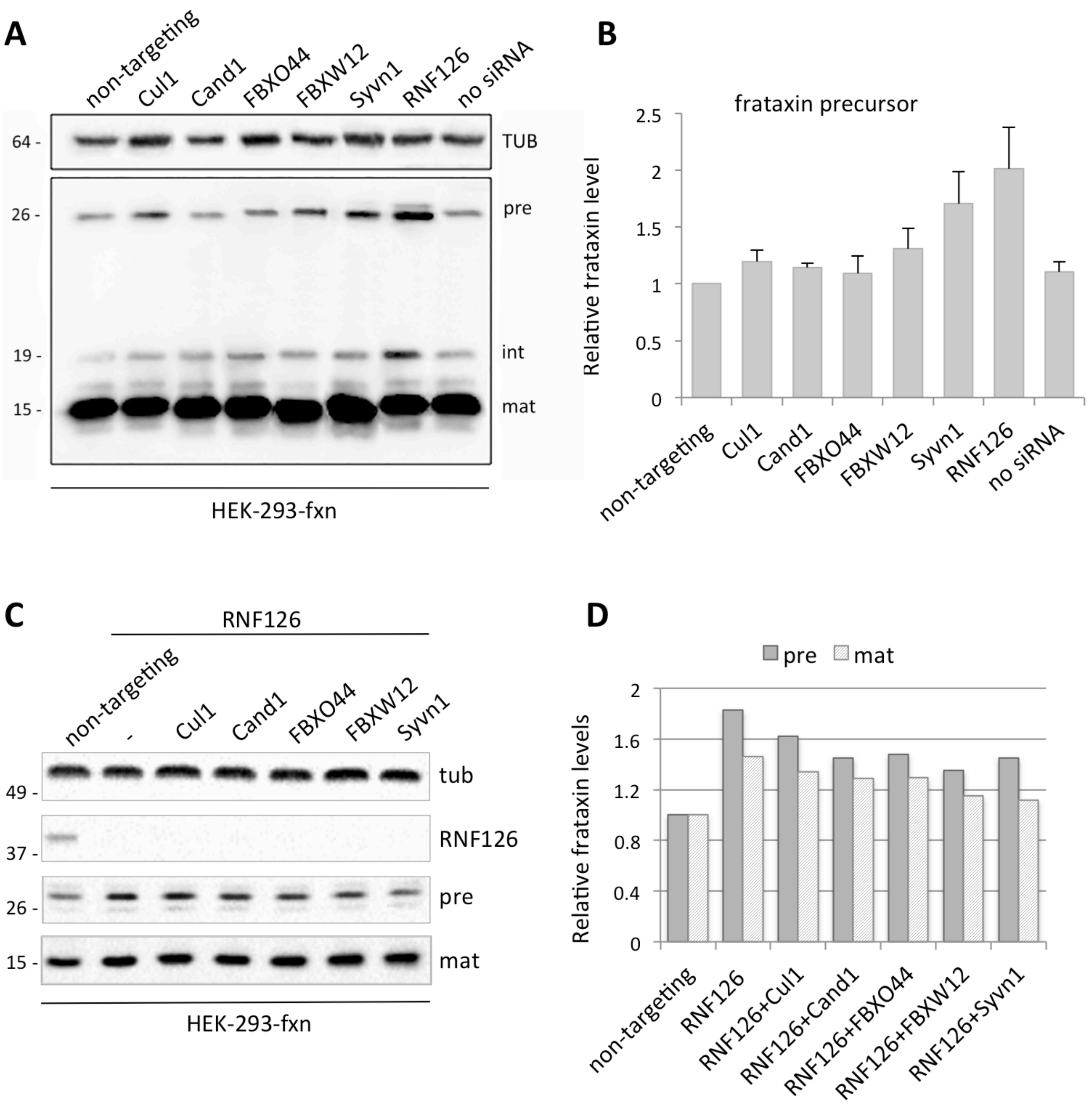

Figure S2. Primary hits validation.

A) siRNAs effect on frataxin levels. HEK-293 cell line stably expressing frataxin$^{1\text{-}210}$ were transfected with the indicated siRNAs. 48 hours after transfection, cells were collected and analyzed by SDS-PAGE and revealed by western blot with anti-frataxin antibody or anti-tubulin. pre: frataxin precursor; int: intermediate frataxin; mat: mature frataxin.

B) The graph represents the relative frataxin precursor levels, quantified as the densitometric ratio between frataxin precursor and tubulin for each lane. Data represent the mean ± 1 S.E.M. from five different independent experiments.

C) Simultaneous knock-down of RNF126 and the other hits identified in the screening does not promote further increase in frataxin levels. HEK-293 cells were transfected with the indicated combination of siRNA and analysed as in A. pre: frataxin

D) The graphs represent relative frataxin precursor (pre) and mature frataxin (mat) abundance as quantitated by densitometric analysis of the blots in C and normalized with tubulin levels. One representative experiment out of two performed with similar results is shown.

Supplemental Figure 3 – Related to Figures 2 and 6

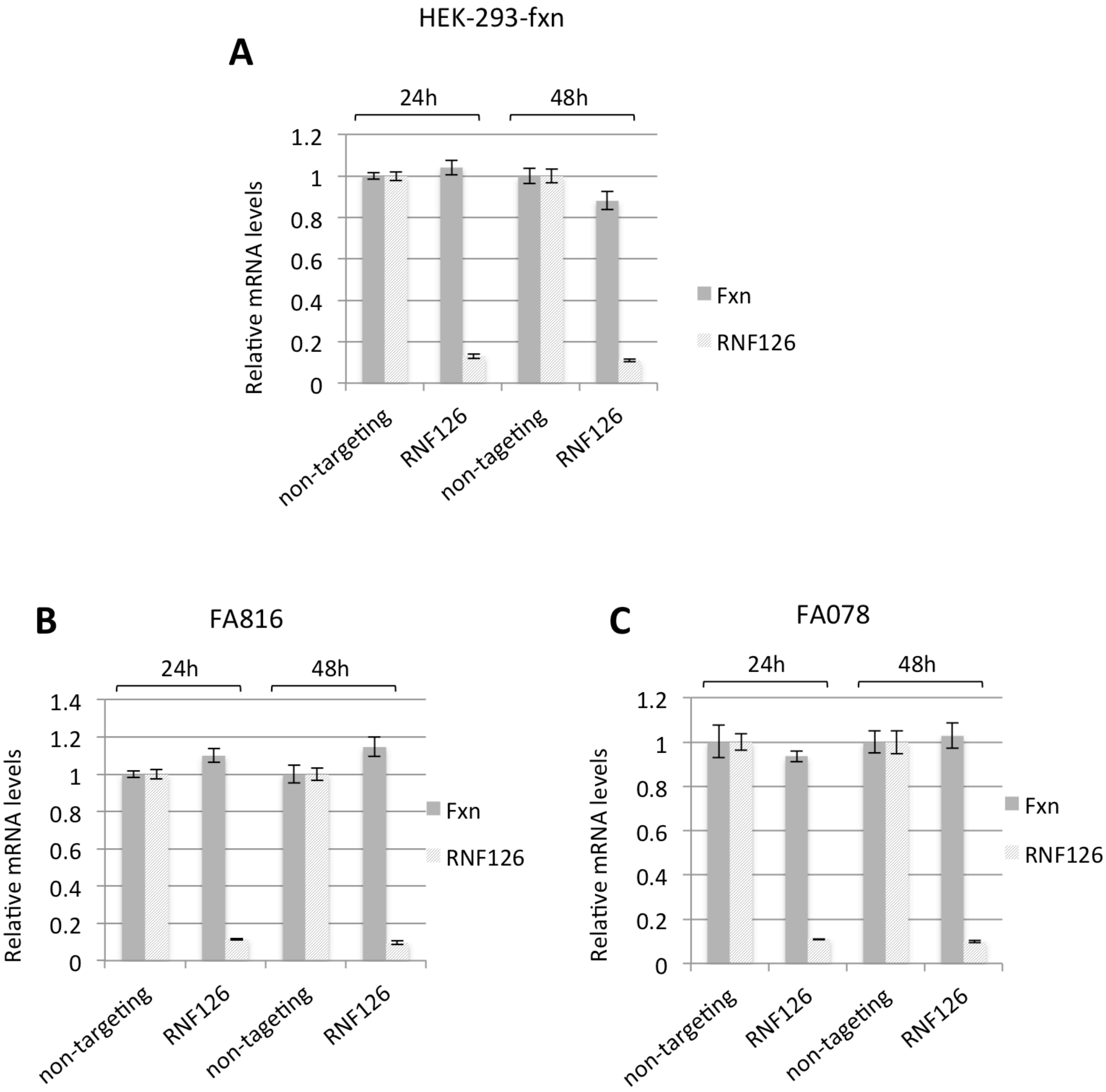

Fig. S3. Silencing of RNF126 E3 ligase does not alter frataxin mRNA levels.
A) 293 Flp-In cell line stably expressing frataxin$^{1-210}$ was transfected with 50 nM of either non-targeting siRNA or RNF126 siRNA, as indicated. Total RNA extracts were collected 24 and 48 hrs post-transfection and analyzed by Real Time PCR. One out of three experiments performed with similar results is shown in the panel. Error bars represent +/- 1SD of the $\Delta\Delta C_T$ value calculated on two technical replicates.
B-C) Fibroblasts derived from 2 different FRDA patients, FA816 (B) and FA078 (C) were transfected with 50 nM of either non-targeting siRNA or RNF126 siRNA, as indicated. Total RNA extracts were collected and analyzed as in A. Error bars represent +/- 1SD of the $\Delta\Delta C_T$ value calculated on two technical replicates.

Supplemental Figure 4 – Related to Figure 2

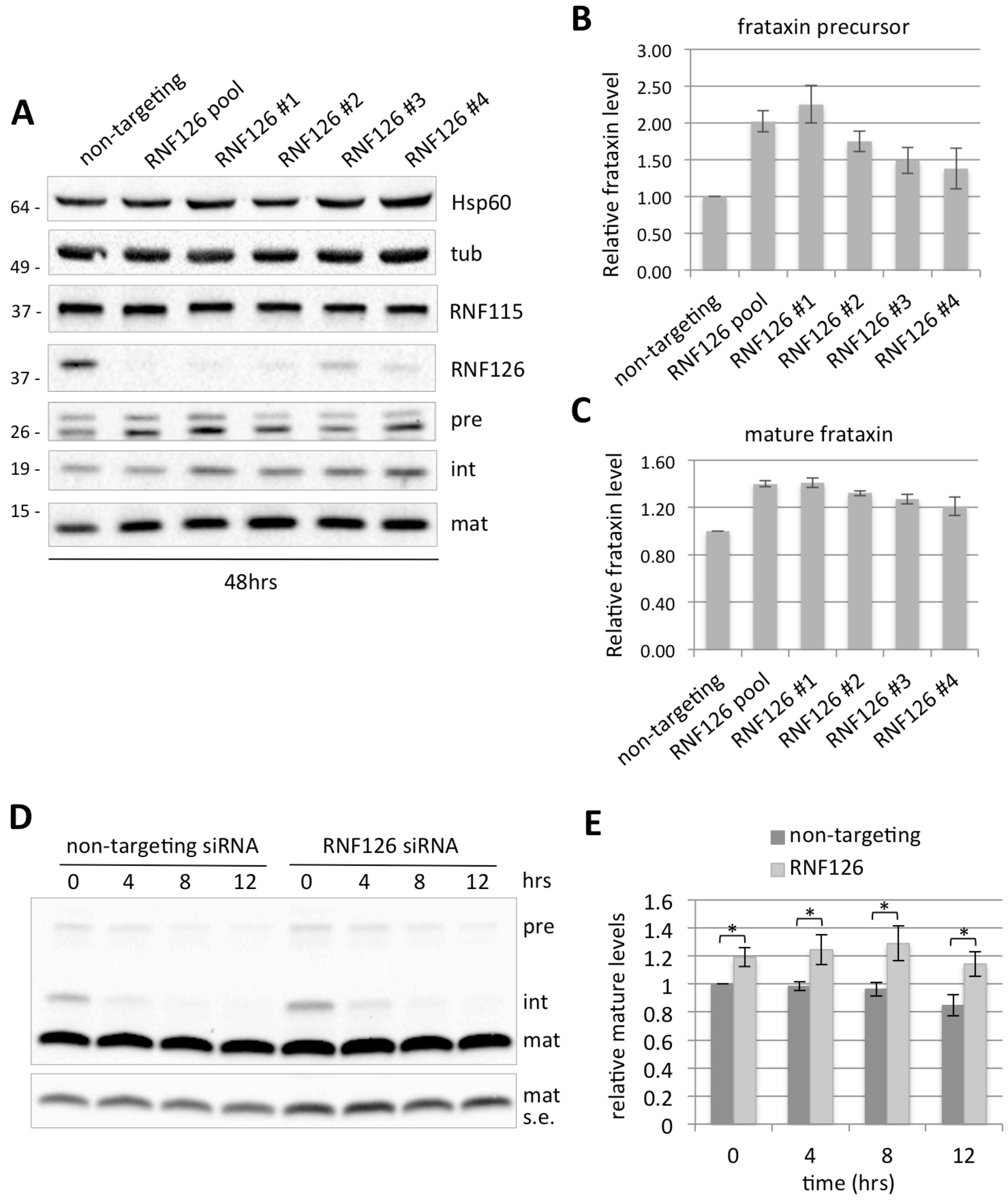

Figure S4. Silencing of RNF126 promotes frataxin accumulation and increases the frataxin precursor stability.

A) Results obtained in Fig 2 A-B using siRNA pools were confirmed with individual siRNA targeting RNF126. 293 Flp-In cell line stably expressing frataxin$^{1-210}$ was transfected with 50 nM of the indicated siRNA pool or 50 nM of the individual RNF126 siRNA. Cell extracts were collected 48 post-transfection and analyzed by WB with anti-frataxin, anti-RNF126, anti-RNF115 and anti HSP60 and anti-tubulin antibody. Pre: precursor; int: intermediate; mat: mature frataxin.

B-C) The graphs represent relative frataxin precursor (B) or mature frataxin (C) abundance as quantitated by densitometric analysis of the blots in A and normalized

with tubulin levels. Data represent the mean ± 1 S.E.M. from three different independent experiments.

D) 293 Flp-In cell line stably expressing frataxin$^{1\text{-}210}$ was transfected with the indicated siRNA pools. 48 hours after transfection cells were treated with 100 nM Actinomycin D for the indicated times. Cell extracts were analyzed by western blot with anti-frataxin antibody. Pre: precursor; int: intermediate; mat: mature frataxin; mat s.e.: mature frataxin, short exposure. E) The graph represents relative mature frataxin abundance as quantitated by densitometric analysis of the blots in A and normalized with tubulin levels. As expected, levels of mature frataxin are increased upon silencing of RNF126. Data represent the mean ± 1 S.E.M. from four different independent experiments. *P*-values were calculated with Student's *t*-test and were statistically significant (*$P<0.05$) compared to non-targeting siRNA-transfected cells.

Supplemental Figure 5 – Related to Figure 3

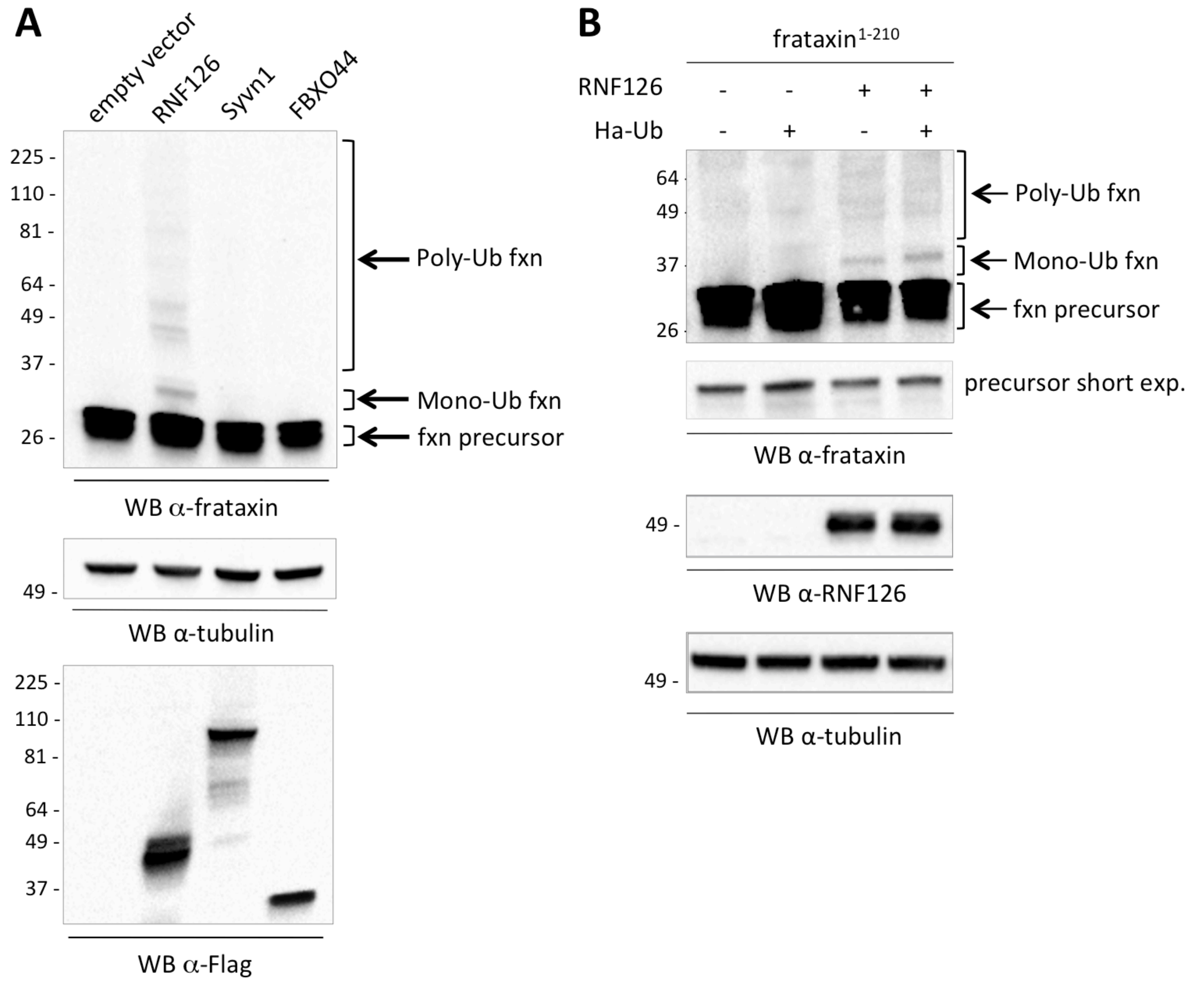

Figure S5. RNF126 promotes frataxin ubiquitination.

A) Expression of RNF126 but not Syvn1 or FBXO44 promotes frataxin ubiquitination. HEK-293 cells were transiently transfected with frataxin$^{1\text{-}210}$, HA-tagged ubiquitin (HA-Ub) and either a control empty vector, or the indicated E3 ligase, expressed as Flag-tagged constructs. Protein extracts were collected 40 hrs post-transfection. Total cell extracts were analyzed by WB with anti-frataxin antibody (upper panel), anti-tubulin, as a loading control (middle panel), or anti-Flag to verify the expression of the E3 ligases (lower panel). Slower-migrating bands can be detected above frataxin precursor, corresponding to mono- and polyubiquitinated frataxin.

B) Expression of RNF126 promotes frataxin ubiquitination in the presence or absence of HA-Ubiquitin. HEK-293 cells were transfected with the indicated constructs. Protein extracts were analyzed as in A with anti-frataxin antibody (upper panel), anti-RNF126 (middle panel) and anti-tubulin, as a loading control (lower panel). Slower-migrating bands above frataxin precursor correspond to mono- and polyubiquitinated frataxin.

Supplemental Figure 6 – Related to Figure 6

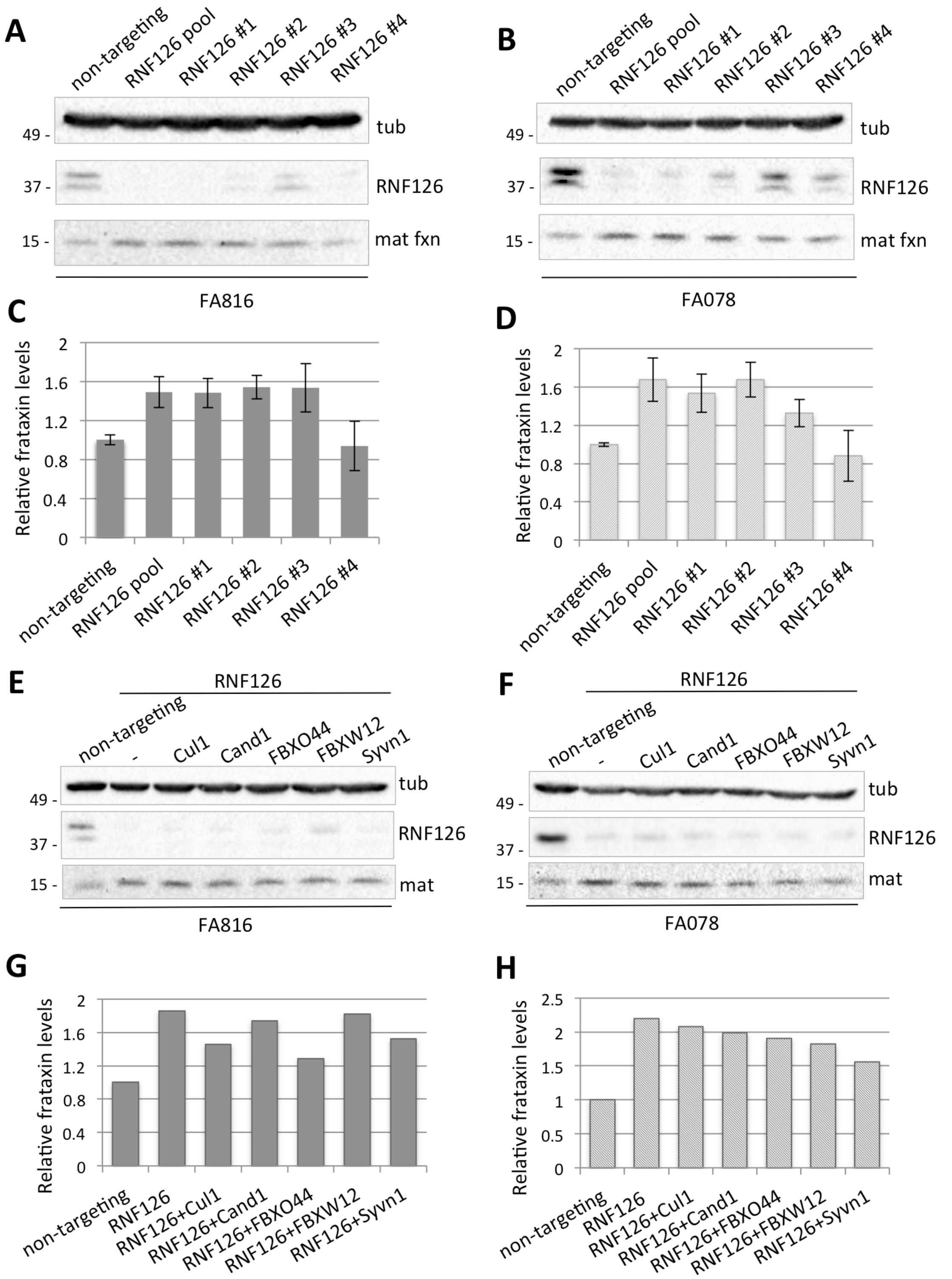

Figure S6. Silencing of RNF126 E3 ligase promotes frataxin accumulation in cells derived from FRDA patients.

A-B) Results obtained in Fig 6 A-B using siRNA pools were confirmed with individual siRNA targeting RNF126. Fibroblasts derived from 2 different FRDA patients, FA816 (A) and FA078 (B) were transfected with 25 nM of the indicated siRNA pool or 25 nM of the individual RNF126 siRNA. Cell extracts were collected 48 hrs post transfection

and analyzed by WB with anti-frataxin antibody, anti-RNF126 and anti-tubulin. Tub: tubulin; mat fxn: mature frataxin.

C-D) The graphs represent relative mature frataxin abundance as quantitated by densitometric analysis of the blots in A and B, respectively and normalized with tubulin levels. Data represent the mean ± 1 S.E.M. from three different independent experiments performed for each patient.

Simultaneous knock-down of RNF126 and the other primary hits does not promote further increase in frataxin levels in cells derived from FRDA patients**:**

E-F) Fibroblasts derived from 2 different FRDA patients, FA816 (E) and FA078 (F) were transfected with the indicated combination of siRNA. Cell extracts were collected 48 hrs post transfection and analyzed by WB with anti-frataxin antibody, anti-RNF126 and anti-tubulin. Tub: tubulin; mat: mature frataxin.

precursor; mat: mature frataxin;

G-H) The graphs represent relative mature frataxin abundance as quantitated by densitometric analysis of the blots in A and B, respectively and normalized with tubulin levels. Data presented are representative of two independent experiments with similar results performed for each patient.

Table S1: siRNA pool target sequences used in this study
Related to Figures 2 and 6, and to Figures S2, S3, S4 (D-E), S6 (E-H).

Gene Name (Catalog Item_Dharmacon)	Target Sequences (5' to 3')	Figure(s)
Non-targeting pool<br>D-001810-10	UGGUUUACAUGUCGACUAA<br>UGGUUUACAUGUUGUGUGA<br>UGGUUUACAUGUUUUCUGA<br>UGGUUUACAUGUUUUCCUA	2, 6, S2, S3, S4 (D-E), S6
CUL1 pool<br>L-004086-00	CAACGAAGAGUUCAGGUUU<br>CGAGGAAGACCGCAAACUA<br>AGACAGUGCUUGAUGUUCA<br>CAUAGAAGACAAAGACGUA	S2, S6 (E-H)
CAND1 pool<br>L-015562-01	GACUUUAGGUUUAUGGCUA<br>CGUGCAACAUGUACAACUA<br>CAACAAGAACCUACAUACA<br>CAUAACAAGCCAUCAUUAA	S2, S6 (E-H)
FBXO44 pool<br>L-019201-00	GAGAGGGCUUCAUCACUGA<br>GCUGAACCCUGACUGGUAA<br>GAAUGGAGGCGAUGAGUGG<br>CAGAUUGCGGGUUCCAAGUA	S2, S6 (E-H)
FBXW12 pool<br>L-032001-00	CAUAUGAGAUCGCAAGUUU<br>GCACAGCCGCAUAACUUUA<br>GGACCAUACUUGUUACUCU<br>GCAAGAGGGUACCAUGAUC	S2, S6 (E-H)
SYVN1 pool<br>L-007090-00	UCAUCAAGGUUCUGCUGUA<br>GAGAAGAGAUGGUGACUGG<br>CAACAUGAACACCCUGUAU<br>GGAAAGGCCUCCAGCUCCU	S2, S6 (E-H)
RNF126 pool<br>L-007015-00	UGUCUAACCUCACCCUCUA<br>CAUCACACAGCUCCUCAAU<br>CGGAUUAUAUCUGUCCAAG<br>GAACAAAACUGCUCCAACA	2, 6, S2, S3, S4 (D-E), S6

Table S2: Individual siRNA target sequences used in this study
Related to Figures 2 and 6 and to Figures S4 (A-C), S6 (A-D).

Gene Name (Catalog Item_Dharmacon)	Target Sequences (5' to 3')	Figure(s)
RNF126 #1<br>J-007015-05	UGUCUAACCUCACCCUCUA	S4 (A-C), S6 (A-D)
RNF126 #2<br>J-007015-06	CAUCACACAGCUCCUCAAU	S4 (A-C), S6 (A-D)
RNF126 #3<br>J-007015-07	CGGAUUAUAUCUGUCCAAG	S4 (A-C), S6 (A-D)
RNF126 #4<br>J-007015-08	GAACAAAACUGCUCCAACA	S4 (A-C), S6 (A-D)
