# Supplementary material for: E3 Ligase RNF126 Directly Ubiquitinates Frataxin, Promoting Its Degradation: Identification of a Potential Therapeutic Target for Friedreich Ataxia
Source: Cell Rep. 2017 Feb 21;18(8):2007–17. doi: 10.1016/j.celrep.2017.01.079 (PMC5329121; doi:10.1016/j.celrep.2017.01.079)
Supplement: Document S2. Article plus Supplemental Information [file mmc2.pdf]

Cell Reports

Article

E3 Ligase RNF126 Directly Ubiquitinates Frataxin, Promoting Its Degradation: Identification of a Potential Therapeutic Target for Friedreich Ataxia

Graphical Abstract

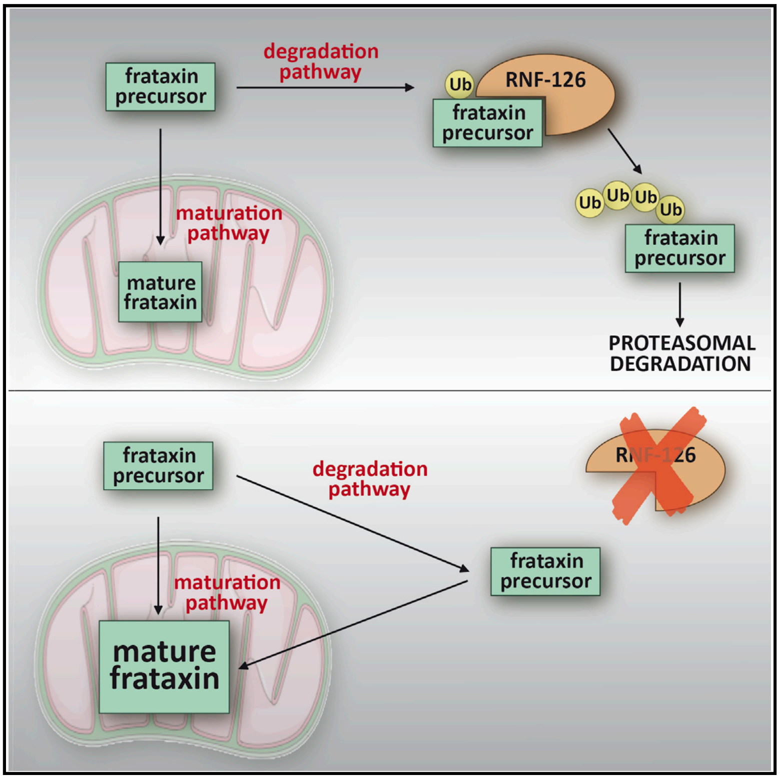

Highlights

- Screening of a siRNA library identifies RNF126 as the frataxin E3 ligase
- RNF126 promotes frataxin ubiquitination and degradation
- RNF126 knockdown induces frataxin accumulation in cells derived from FRDA patients
- RNF126 represents a potential therapeutic target for Friedreich ataxia

Authors

Monica Benini, Silvia Fortuni, Ivano Condò, ..., Gaetano Arcuri, Roberto Testi, Alessandra Rufini

Correspondence

rufini@med.uniroma2.it

In Brief

Friedreich ataxia is caused by insufficient levels of frataxin. Through the functional screening of a siRNA library, Benini et al. identified RNF126 as the E3 ligase that ubiquitinates frataxin and promotes its degradation. siRNA-mediated knockdown of RNF126 increases frataxin levels in cells derived from patients, unveiling its potential therapeutic relevance.

CrossMark

Benini et al., 2017, Cell Reports *18*, 2007–2017
February 21, 2017 © 2017 The Author(s).
http://dx.doi.org/10.1016/j.celrep.2017.01.079

CellPress

Cell Reports
Article

OPEN ACCESS
CellPress

E3 Ligase RNF126 Directly Ubiquitinates Frataxin, Promoting Its Degradation: Identification of a Potential Therapeutic Target for Friedreich Ataxia

Monica Benini,[1,2] Silvia Fortuni,[1] Ivano Condò,[1] Giulia Alfedi,[1] Florence Malisan,[1] Nicola Toschi,[3,4] Dario Serio,[1,2] Damiano Sergio Massaro,[1] Gaetano Arcuri,[1] Roberto Testi,[1,2] and Alessandra Rufini[1,2,5,*]
[1]Laboratory of Signal Transduction, Department of Biomedicine and Prevention, University of Rome "Tor Vergata," Via Montpellier 1, 00133 Rome, Italy
[2]Fratagene Therapeutics Srl, Viale dei Campioni 8, 00144 Rome, Italy
[3]Medical Physics Section, Department of Biomedicine and Prevention, University of Rome "Tor Vergata," Via Montpellier 1, 00133 Rome, Italy
[4]Department of Radiology, Athinoula A. Martinos Center for Biomedical Imaging and Harvard Medical School, Boston, MA 02115, USA
[5]Lead Contact
*Correspondence: rufini@med.uniroma2.it
http://dx.doi.org/10.1016/j.celrep.2017.01.079

SUMMARY

Friedreich ataxia (FRDA) is a severe genetic neurodegenerative disease caused by reduced expression of the mitochondrial protein frataxin. To date, there is no therapy to treat this condition. The amount of residual frataxin critically affects the severity of the disease; thus, attempts to restore physiological frataxin levels are considered therapeutically relevant. Frataxin levels are controlled by the ubiquitin-proteasome system; therefore, inhibition of the frataxin E3 ligase may represent a strategy to achieve an increase in frataxin levels. Here, we report the identification of the RING E3 ligase RNF126 as the enzyme that specifically mediates frataxin ubiquitination and targets it for degradation. RNF126 interacts with frataxin and promotes its ubiquitination in a catalytic activity-dependent manner, both in vivo and in vitro. Most importantly, RNF126 depletion results in frataxin accumulation in cells derived from FRDA patients, highlighting the relevance of RNF126 as a new therapeutic target for Friedreich ataxia.

INTRODUCTION

Friedreich ataxia (FRDA, OMIM: 229300) is a debilitating, life-shortening, neurodegenerative disorder affecting mainly the nervous system and the heart. It is classified as a rare disease; however, it is the most common form of inherited ataxia, with an estimated prevalence of 1 in 50,000 individuals in the Caucasian population (Alper and Narayanan, 2003; Delatycki et al., 2000). Symptoms are progressive and usually begin around puberty, although late-onset cases have been described. Early signs include sensory deficit with consequent loss of movement coordination and gait ataxia. Patients are usually wheelchair bound within 15 years after diagnosis and require assistance to accomplish daily activities (Collins, 2013). Other hallmarks of disease progression include visual impairment because of optic nerve atrophy, dysarthria, and dysphagia. Skeletal abnormalities such as scoliosis and pes cavus are also present. Patients develop a hypertrophic cardiomyopathy that often leads to premature death (Koeppen et al., 2015; Lane et al., 2013; Weidemann et al., 2013). Moreover, about 25% of patients develop diabetes mellitus (Cnop et al., 2013; Igoillo-Esteve et al., 2015). Neurological symptoms are caused by degeneration of sensory neurons in the dorsal root ganglia and in the dentate nucleus of the cerebellum (Koeppen and Mazurkiewicz, 2013). So far, there is no approved therapy for Friedreich ataxia, and only palliative treatments are available for patients. Identification of a treatment for Friedreich ataxia represents a major unmet medical need (Evans-Galea et al., 2014; Strawser et al., 2014; Wilson, 2012).

The disease is caused by reduced expression of the essential mitochondrial protein frataxin (Campuzano et al., 1997). The underlying mutation primarily consists of a homozygous trinucleotide guanine-adenine-adenine (GAA) repeat expansion within the first intron of the corresponding gene (Campuzano et al., 1996). The presence of the GAA tract severely impairs transcription initiation (Chutake et al., 2014a) and elongation (Kim et al., 2011; Li et al., 2015) of the frataxin gene, mainly due to formation of an atypical triplex non-B DNA structure (sticky DNA) (Vetcher et al., 2002) or DNA-RNA hybrids (R loops) (Grabczyk et al., 2007; Groh et al., 2014) and epigenetic modifications that induce a non-permissive chromatin conformation of this DNA region (Al-Mahdawi et al., 2008; Herman et al., 2006), resulting in reduced levels of frataxin protein. Patients live with 5%–30% residual frataxin, the severity of the disease being correlated to the extent of frataxin reduction, which in turn correlates to the length of the GAA expansion (Chutake et al., 2014b). Thus, any increase in frataxin levels is considered therapeutically relevant. Strategies to cure Friedreich ataxia are therefore mostly aimed at increasing the amount of frataxin in patients' cells (Soragni et al., 2014; Wilson, 2012). Frataxin is a nuclear-encoded mitochondrial protein that plays a crucial role in the biosynthesis of iron-sulfur clusters (Bulteau et al., 2004; Tsai and Barondeau, 2010; Vaubel and Isaya, 2013) and in iron metabolism (Anzovino et al., 2014; Richardson et al., 2010). Iron-sulfur clusters are

CrossMark

This is an open access article under the CC BY license (http://creativecommons.org/licenses/by/4.0/).

important cofactors required for proper functioning of enzymes such as aconitase and complexes I, II, and III of the mitochondrial electron transport chain (Rouault and Tong, 2008). Frataxin deficiency therefore results in defective aconitase activity (Condò et al., 2010), impaired mitochondrial respiration, reduced ATP production, imbalance of iron metabolism, mitochondrial iron overload, and increased sensitivity to oxidative stress (Martelli and Puccio, 2014; Pastore and Puccio, 2013). These events are eventually responsible for neuronal degeneration, particularly in the dorsal root ganglia (Koeppen and Mazurkiewicz, 2013; Lynch et al., 2012).

Frataxin is produced in the cytosol as a precursor form with an N-terminal mitochondrial localization signal, which allows the precursor to be directed to mitochondria. During mitochondrial import, the precursor undergoes a two-step catalytic processing that, through the generation of an intermediate form, yields the mature functional form of frataxin (Condò et al., 2007), which is localized in the mitochondrial matrix. We have previously shown that during the normal maturation process, a significant amount of frataxin precursor is degraded through the ubiquitin (Ub)-proteasome pathway, before its mitochondrial import (Rufini et al., 2011). Most current therapeutic approaches aim at promoting frataxin gene transcription (Strawser et al., 2014); however, molecular characterization of the frataxin degradation pathway suggested the possibility to increase frataxin protein by preventing its degradation. We have identified small molecules named ubiquitin-competing molecules that, by docking on the frataxin ubiquitination site, prevent frataxin precursor degradation and eventually promote accumulation of functional mature frataxin (Rufini et al., 2011, 2015). These molecules provide the rational basis for the development of therapeutic approaches that aim at preventing frataxin degradation. Another strategy to prevent frataxin degradation could be the inhibition of the enzyme responsible for frataxin ubiquitination. Protein ubiquitination is a finely regulated process that ensures tight control of intracellular proteins levels, in particular through the ability of E3 ligase enzymes to selectively recognize their substrates. E3 ligases are therefore considered attractive targets for the development of specific therapies. However, the E3 ligase that specifically recognizes frataxin and targets it for degradation was unknown.

Here we report on the identification of really interesting new gene (RING) finger protein 126 (RNF126) as the E3 ligase that ubiquitinates frataxin. We show that RNF126 interacts with frataxin and directly promotes its ubiquitination, both in vitro and in vivo. We show that RNF126 knockdown results in frataxin accumulation in cells derived from FRDA patients, suggesting the therapeutic potential of strategies aimed at inhibiting RNF126. This enzyme therefore represents a novel important therapeutic target for Friedreich ataxia.

RESULTS

Identification of the Frataxin-Specific E3 Ligase

To identify the E3 ligase responsible for frataxin ubiquitination, we performed a functional screening of a small interfering RNA (siRNA) library targeting more than 600 cellular E3 ligases. By preventing the ubiquitin-dependent degradation of frataxin, the silencing of the critical E3 ligase is expected to yield an increase in frataxin abundance. To measure variation in frataxin levels upon siRNA transfection, we generated a fusion construct between frataxin and ProLabel that was used as a reporter in a cell-based assay. The system is based on β-galactosidase enzyme fragment complementation (Eglen, 2002). The 6 kDa ProLabel tag encodes the inactive α fragment of the β-galactosidase enzyme. When the Ω subunit of the enzyme is added, together with the substrate, the two subunits form an active enzyme that generates a chemiluminescent signal whose intensity correlates to the amount of frataxin-ProLabel fusion present in the cells (Figure 1A). Regular processing of the frataxin precursor into intermediate and mature forms in the ProLabel fusion construct was verified by western blot analysis of transfected cells (Figure S1A). To validate this system, cells transfected with frataxin-ProLabel were treated with the proteasome inhibitor MG132, as a positive control. As previously reported (Rufini et al., 2011), accumulation of the frataxin precursor can be observed 24 hr after MG132 treatment. In this case, up to an 8-fold increase (5.37 ± 2.52) in the intensity of the luminescence signal could be detected by luminometer reading, confirming the sensitivity and the wide dynamic range of the system. The Z' value for the assay was 0.4, indicating an acceptable to good assay (Birmingham et al., 2009). The library consisted of pools of four siRNAs per gene prearrayed into nine 96-wells plates (Figure S1B). HEK293 cells transiently transfected with frataxin-ProLabel were reverse transfected with the siRNA library. Then, 48 hr after siRNA transfection, the luminescence signal was assessed by luminometer reading, upon addition of the complementary β-galactosidase subunit and substrate (Figure 1B). The increase in the luminescence signal by siRNA transfection was most likely due to suppression of the expression of the critical E3 ligase. Six replicates of each plate were screened independently. From the screening, we isolated six candidate genes potentially involved in the regulation of frataxin stability that were selected for further validation (Figures S1C and S1D).

Depletion of RNF126 Increases Frataxin Stability and Promotes Frataxin Accumulation

The six candidate genes identified in the screening were individually validated in the HEK293 Flp-In cell line stably overexpressing frataxin$^{1-210}$ to evaluate their ability to increase frataxin levels (Figures S2A and S2B). This cell line has a single extra copy of the frataxin coding sequence integrated into the genome, allowing detection of frataxin precursor while maintaining frataxin products at near-physiological levels. We have previously shown that frataxin precursor readily accumulates in cells when frataxin degradation is prevented, and it is subsequently converted into the mature form (Rufini et al., 2011). These cells enable the detection of precursor, intermediate, and mature frataxin, thus allowing rapid evaluation of siRNA efficacy. Among the six candidate genes identified, we observed that silencing of the RING E3 ubiquitin ligase RNF126 consistently promotes an increase in frataxin levels. As shown in Figures 2A–2C, transfection of HEK293 cells with the specific siRNA pool efficiently suppresses RNF126 expression and promotes increase in both frataxin precursor and mature frataxin, while silencing of a non-related E3 ligase has no effect. No significant alteration on frataxin mRNA levels was observed upon RNF126 knockdown (Figure S3A).

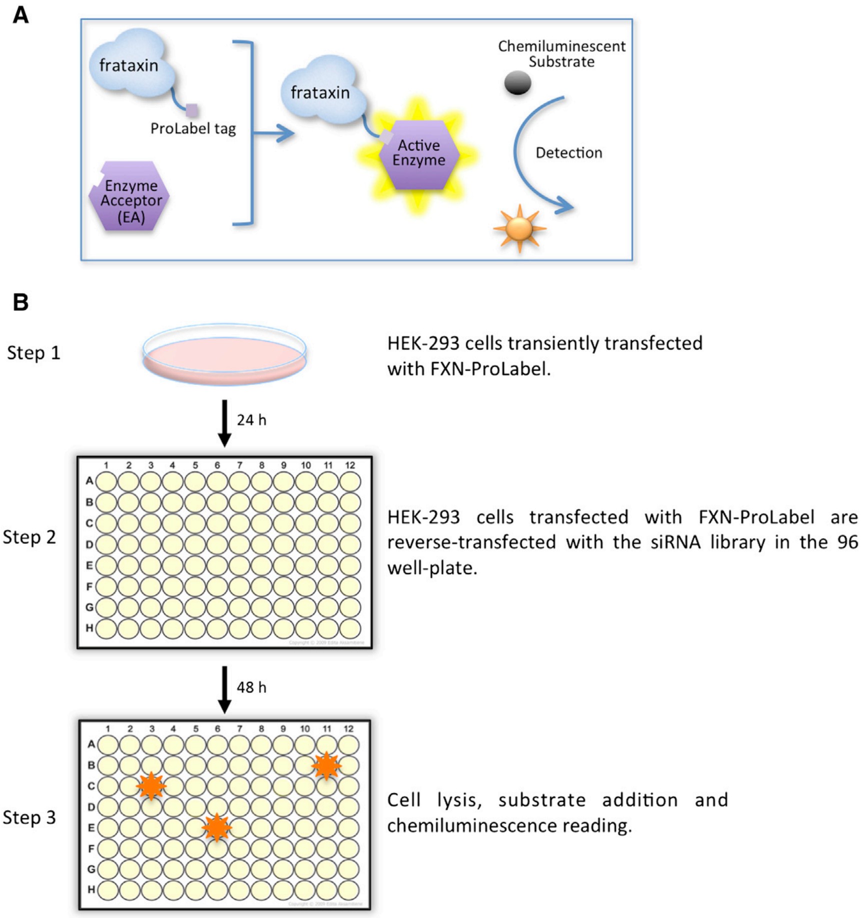

Figure 1. Schematic Representation of the siRNA Screening Assay and Workflow

(A) Schematic representation of the frataxin-ProLabel fusion construct used in the screening and the enzyme fragment complementation assay. The 6 kDa ProLabel tag encodes the inactive α fragment of the β-galactosidase enzyme, and it is expressed as a C-terminal tag fused to frataxin. The enzyme acceptor (EA) represents the complementary part of the enzyme. When the EA is added to the cells, together with the substrate, the two subunits combine to form a complete, active β-galactosidase enzyme that cleaves the chemiluminescent substrate, generating a luminescent signal proportional to the amount of frataxin-ProLabel.

(B) Schematic representation of the screening workflow.

We considered the possibility that more than one E3 ligase is involved in the regulation of frataxin turnover; however, simultaneous knockdown of RNF126, together with the primary hits, does not result in additional increase in frataxin precursor compared to RNF126 silencing alone (Figures S2C and S2D). Results obtained with the pool of four siRNAs targeting RNF126 were confirmed in cells transfected with individual RNF126 siRNA (Figures S4A–S4C). Moreover, we verified that silencing of RNF126 did not affect the expression of the closely related E3 ligase RNF115, which shares a high degree of homology with RNF126 (Figure S4A) (Krysztofinska et al., 2016). Depletion of RNF126 by specific siRNA delays the degradation of frataxin precursor and increases its stability. When protein synthesis is blocked by actinomycin D, frataxin precursor has a longer half-life in cells transfected with RNF126 siRNA compared to cells transfected with a control siRNA (Figures 2D and 2E; Figures S4D and S4E). These data indicate that RNF126 is responsible for the degradation of frataxin precursor and therefore is involved in the control of frataxin levels.

RNF126 Triggers Frataxin Ubiquitination

To further investigate the contribution of RNF126 in the control of frataxin degradation, we tested its ability to promote frataxin ubiquitination. Overexpression of RNF126 in HEK293 cells, but not of its catalytically inactive mutant, promotes frataxin ubiquitination. As shown in Figure 3A, when cells are cotransfected with frataxin and hemagglutinin (HA)-tagged ubiquitin (HA-Ub), together with RNF126, mono- and polyubiquitinated forms of frataxin can be detected as slower-migrating bands above the frataxin precursor on a western blot with anti-frataxin antibody. Ubiquitinated forms of frataxin can also be immunoprecipitated with anti-HA antibody and detected with anti-frataxin, confirming that ubiquitinated forms of frataxin are present when cells are transfected with RNF126 (Figure 3B). To confirm the requirement for the E3 ubiquitin ligase catalytic activity of RNF126 for the ubiquitination of frataxin, we generated a catalytically inactive version of RNF126. RNF126 contains an N-terminal zinc-finger domain and a C-terminal RING finger domain, which is implicated in its ubiquitin ligase activity. The catalytically inactive mutant, RNF126$^{C229/232A}$, was generated by substituting the two critical cysteines in the C-terminal RING domain of RNF126 with two alanines, yielding an inactive enzyme (Zhi et al., 2013). Ubiquitinated frataxin forms were not detected when cells were transfected with RNF126$^{C229/232A}$. These data indicate that RNF126 can trigger frataxin ubiquitination and that this is dependent on its catalytic activity. To rule out the possible contribution of the other identified hits in the ubiquitination of frataxin precursor, we investigated the ability of two other hits, namely, Syvn1 and FBXO44, to promote frataxin ubiquitination in the same experimental setting. However, no significant impact on frataxin ubiquitination was observed upon transfection of either Syvn1 or FBXO44, supporting the specificity of the ubiquitination promoted by RNF126 (Figure S5A). Moreover, RNF126 induces frataxin ubiquitination also in the absence of HA-Ub overexpression (Figure S5B).

Frataxin Interacts with Its E3 Ligase RNF126

To validate the hypothesis that the RNF126 can directly ubiquitinate frataxin, we investigated whether the two proteins directly

OPEN ACCESS
CellPress

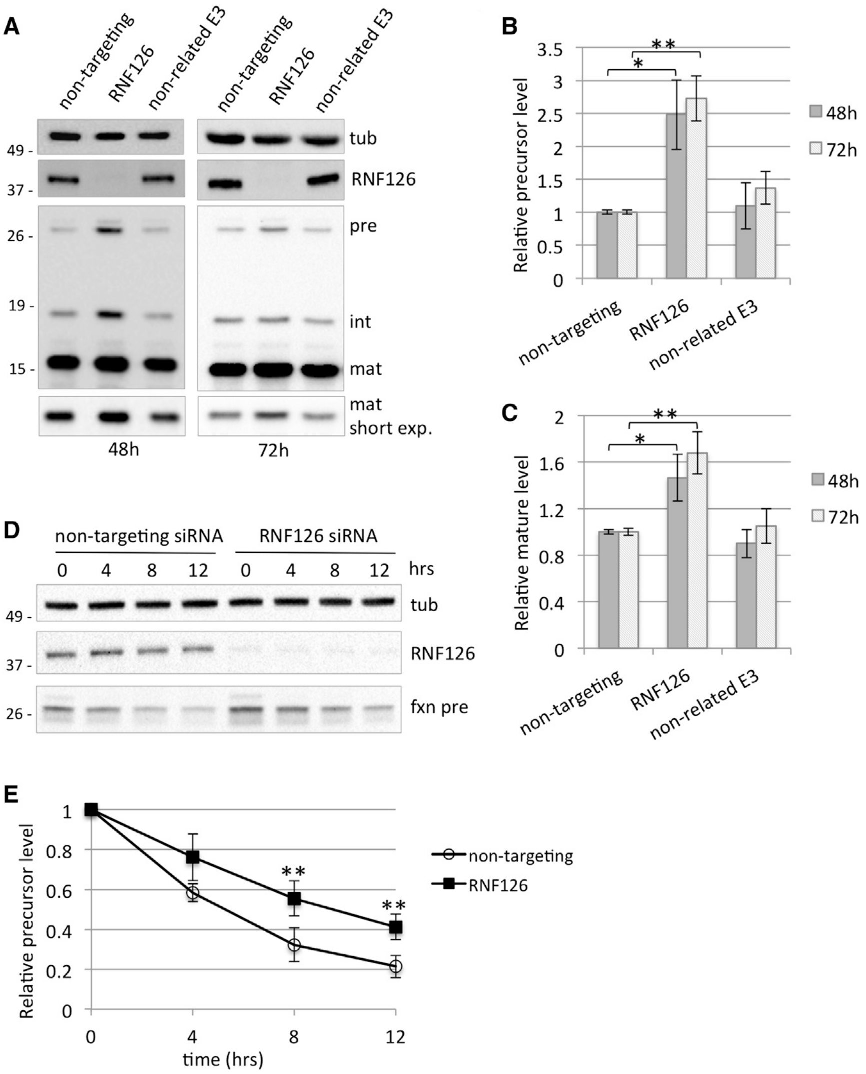

Figure 2. Silencing of RNF126 Promotes Accumulation of Frataxin Precursor and Mature Forms by Preventing Its Degradation

(A) 293 Flp-In cell line stably expressing frataxin$^{1–210}$ was transfected with the indicated siRNA pools. Cell extracts were collected 48 or 72 hr post-transfection and analyzed by WB with anti-frataxin, anti-RNF126, and anti-tubulin antibody. Pre, precursor; int, intermediate; mat, mature frataxin.

(B and C) Graphs representing relative frataxin precursor (B) or mature frataxin (C) abundance as quantitated by densitometric analysis of the blots in (A) and normalized with tubulin levels.

(D) 293 Flp-In cell line stably expressing frataxin$^{1–210}$ was transfected with the indicated siRNA pools. Then, 48 hr after transfection, cells were treated with 100 nM actinomycin D for the indicated times. Cell extracts were analyzed by western blot with anti-frataxin, anti-RNF126, and anti-tubulin antibody.

(E) Graph representing relative frataxin precursor abundance as quantitated by densitometric analysis of the blot in (D) and normalized with tubulin levels.

Data represent the mean ± 1 SEM from four independent experiments. The p values were calculated with Student's t test and were statistically significant (**$p < 0.01$) compared to non-targeting siRNA-transfected cells.

interact with each other. To this aim, we cotransfected HEK293 cells with frataxin and RNF126, followed by immunoprecipitation with anti-frataxin antibody. As shown in Figure 4A, RNF126 can be detected in the immunoprecipitated proteins, indicating that it can form a complex with frataxin. Conversely, when we immunoprecipitated RNF126 from the cell extract, we could detect the frataxin precursor associated with it (Figure 4B). Although the precursor is not the most abundant form of frataxin that is present in the cell, it is preferentially coprecipitated with RNF126, compared to the intermediate and mature forms of frataxin. These data are in agreement with the notion that the frataxin precursor is ubiquitinated before its mitochondrial import and maturation, and they suggest that the N-terminal portion of frataxin is required for interaction with the E3 ligase. In addition, the frataxin precursor can be coprecipitated with the endogenous RNF126 (Figure 4B, lane 3). Slower migrating bands above the frataxin precursor, detected by the anti-frataxin antibody, are also coprecipitated with RNF126, indicating that ubiquitinated frataxin can be found associated with RNF126 (Figure 4B, lane 4). Although the frataxin precursor is found associated with the inactive mutant of RNF126, no ubiquitinated frataxin is detected as being associated with it (Figure 4B, lane 6).

RNF126 Promotes Frataxin Ubiquitination In Vitro

To confirm the ability of RNF126 to directly ubiquitinate frataxin, we established an in vitro assay with purified recombinant components. In this system, RNF126 can catalyze both mono- and polyubiquitination of frataxin in a dose-dependent manner. The ubiquitination pattern observed in this in vitro assay is similar to that observed in the in-cell ubiquitination assay (Figure 3A). Frataxin ubiquitination only occurs when the E1 ubiquitin-activating enzyme, which is required to activate the ubiquitination process, and the E2 ubiquitin-conjugating enzyme are present in the assay mixture, together with ubiquitin and ATP. These data indicate that when all components of the ubiquitination cascade are present, the E3 ligase RNF126 is sufficient to promote frataxin ubiquitination in vitro and does not require additional cofactors (Figure 5A). UbcH5b was described as the preferred E2 for RNF126 (Delker et al., 2013; Smith et al., 2013) and was therefore used in these assays. However, to confirm the specific requirement of UbcH5b as the E2 in this reaction, we tested the ability of a different E2, UbcH13/Mms2, to cooperate with RNF126 in the ubiquitination of frataxin. As shown in Figure 5B, only UbcH5b can promote frataxin ubiquitination, suggesting that RNF126 specifically requires the

OPEN ACCESS
CellPress

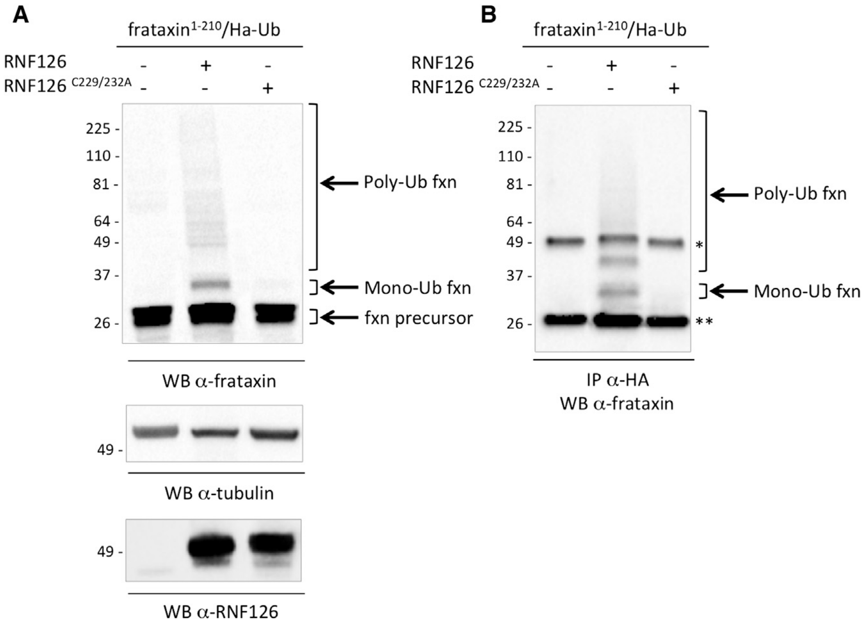

Figure 3. Expression of RNF126, But Not Its Catalytically Inactive Mutant, Promotes Frataxin Ubiquitination

(A) HEK293 cells were transiently transfected with frataxin$^{1–210}$, HA-tagged ubiquitin (HA-Ub), and a control empty vector, RNF126, or its catalytically inactive mutant. Protein extracts were collected 40 hr post-transfection. Total cell extracts were analyzed by WB with anti-frataxin antibody or anti-tubulin as a loading control. Slower-migrating bands can be detected above the frataxin precursor, corresponding to mono- and poly-ubiquitinated frataxin.

(B) Cells were transfected as in (A). Mono- and polyubiquitin-conjugated forms of frataxin can be detected by WB with anti-frataxin antibody on anti-HA immunoprecipitates. Control samples (excluded from these panels) were treated with 10 μM of the proteasome inhibitor MG132 as a positive control for the detection of ubiquitinated frataxin.

*Antibody heavy chain; **antibody light chain.

E2 UbcH5b to catalyze frataxin ubiquitination. RNF126 ubiquitin ligase activity depends on its N-terminal zinc-finger domain and its C-terminal RING domain (Smith et al., 2013). The Zn-chelating compound 1,10-phenanthroline is therefore expected to perturb the structure of RNF126, likely disrupting its catalytic activity. We could show that 1,10-phenanthroline can prevent frataxin ubiquitination in a dose-dependent way, confirming that frataxin ubiquitination is dependent on the RNF126 E3 ligase activity (Figure 5C).

RNF126 Depletion Promotes Frataxin Accumulation in FRDA Patient Cells

Our experimental evidence suggests that RNF126 is the E3 ligase responsible for frataxin ubiquitination; therefore, the silencing of the corresponding gene impairs frataxin degradation and results in an increase in frataxin levels (Figure 2). To determine the effect of this mechanism in a disease-relevant cell model, we used primary fibroblasts derived from FRDA patients and tested the effect of siRNA-mediated knockdown of RNF126 expression. As shown in Figure 6, transfection with the specific siRNA reduces the expression of RNF126 and promotes accumulation of mature frataxin in fibroblasts derived from two patients, without affecting frataxin gene transcription (Figures S3B and S3C). Data obtained with transfection of a pool of four siRNA targeting RNF126 were confirmed with individual siRNA for RNF126 (Figures S6A–S6D). Silencing of RNF126, together with the other primary hits identified in the screening, does not contribute to frataxin upregulation in either of the two patient-derived fibroblasts (Figures S6E–S6H). Collectively, these data indicate that the E3 ligase RNF126 controls frataxin abundance in primary cells derived from patients and suggest that inhibition of RNF126 may represent a new therapeutic strategy to achieve an increase in frataxin levels.

DISCUSSION

The ubiquitin-proteasome system (UPS) represents the most important cellular mechanism for regulated protein degradation (Ciechanover, 2013). Modification of a protein by the covalent attachment of one or more ubiquitin moieties determines the fate of the protein and therefore has important biological consequences (Popovic et al., 2014; Schmidt and Finley, 2014). Modulation of the UPS pathway for therapeutic intervention represents a field of growing interest. Ubiquitination of a target substrate is achieved through the concerted and sequential action of three enzymes: the E1 ubiquitin-activating enzyme, an E2 ubiquitin-conjugating enzyme, and an E3 ubiquitin ligase (Nagy and Dikic, 2010). The E3 ligase is the enzyme that mediates substrate recognition and thus is responsible for selectivity and specificity of the system (Berndsen and Wolberger, 2014). Targeting the E3 ligase is therefore the most specific and desirable approach to modulate protein ubiquitination. E3 ligases represent key targets for drug development (Cohen and Tcherpakov, 2010; Lill and Wertz, 2014; Zhang and Sidhu, 2014).

Presently, no effective therapy has been approved to treat FRDA. Current strategies aim to increase frataxin expression or to intervene in the pathogenic cascade downstream of frataxin deficiency. FRDA patients live with a reduced and insufficient amount of frataxin protein; thus, the main goal of a specific therapy for Friedreich ataxia would be to restore physiological frataxin levels. One possible therapeutic approach to achieve an increase in frataxin levels is to prevent the UPS-mediated degradation of frataxin precursor, thus allowing more frataxin to be imported and processed into mitochondria to generate the mature functional form of frataxin (Rufini et al., 2011, 2015). The inhibition of the E3 ligase responsible for frataxin ubiquitination could represent therefore an attractive therapeutic strategy to prevent frataxin degradation. The frataxin-specific E3 ligase has been unknown.

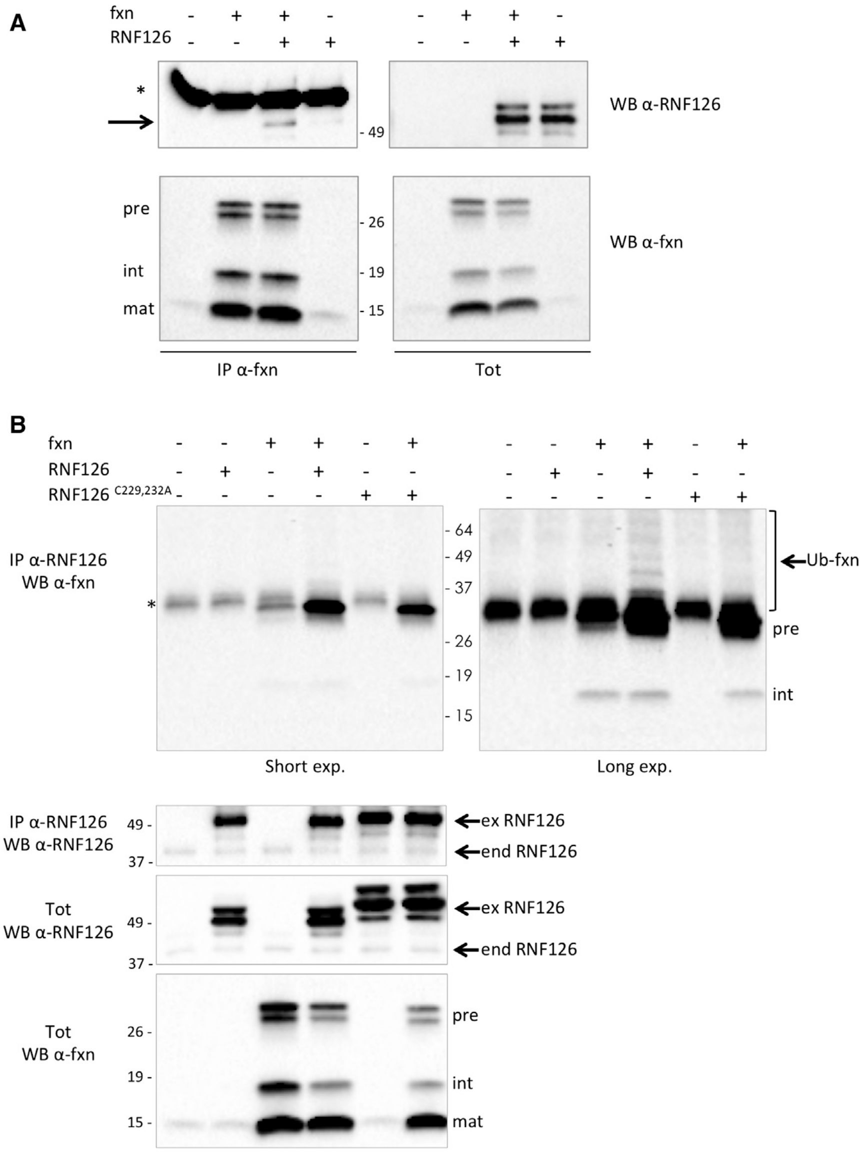

Figure 4. Frataxin Precursor Interacts with RNF126

HEK293 cells were transiently transfected with the indicated constructs. Protein extracts were collected 40 hr post-transfection.

(A) Proteins were immunoprecipitated with anti-frataxin (anti-fxn) antibody. Arrow indicates RNF126 coprecipitated with frataxin. *Antibody heavy chain.

(B) Proteins were immunoprecipitated with anti-RNF126 antibody. Upper left panel: short exposure. Upper right panel: long exposure. *Nonspecific band.

Immunoprecipitate (IP) and total cell extract (tot) were probed on a WB with anti-frataxin (anti-fxn) antibody or anti-RNF126 antibody, as indicated. Pre, precursor int, intermediate; mat, mature frataxin; Ub-fxn, ubiquitinated frataxin; ex RNF126, overexpressed exogenous RNF126; end RNF126, endogenous RNF126.

Through a screening of more than 600 E3 ligases, we report here the identification of RNF126 as the E3 ubiquitin ligase that specifically ubiquitinates frataxin precursor and targets it for proteasomal degradation. Although we cannot exclude that under particular circumstances additional E3 ligases contribute to the regulation of frataxin degradation, the other hits that we identified in the screening do not seem to contribute to this process in a significant manner (Figures S2 and S6). Here we show that by regulating the amount of frataxin precursor available for mitochondrial import, RNF126 eventually controls the levels of mature frataxin. Depletion of RNF126 in cells derived from FRDA patients promotes accumulation of mature frataxin, pointing toward this E3 ligase as a new potential therapeutic target for Friedreich ataxia. Although no pharmacological reference is available to determine a therapeutically relevant threshold of frataxin increase, it was previously shown that a modest increase in frataxin levels is sufficient to induce a functional rescue of aconitase activity in cells derived from patients (Rufini et al., 2011, 2015).

RNF126 belongs to the RING type family of E3 ubiquitin ligases, which are characterized by the presence of a RING (really interesting new gene) domain that binds the E2 ubiquitin-conjugating enzyme and catalyzes the transfer of ubiquitin from the E2 to the substrate (Deshaies and Joazeiro, 2009; Metzger et al., 2014). In particular, RNF126 contains an N-terminal zinc-finger domain and a C-terminal RING finger domain. The N-terminal zinc-finger domain was shown to directly interact with ubiquitin, while the C-terminal RING domain was shown to be required for its E3 ubiquitin ligase activity (Smith et al., 2013). E3 ligases are often described as directly interacting with their specific substrates. We could show an association between RNF126 and frataxin precursor in cells. Substitution of two critical cysteines in the RING domain abrogates the catalytic activity of RNF126 (Zhi et al., 2013). Although the mutant retains the ability to interact with frataxin, the mutation in the RING domain abolishes its ability to ubiquitinate frataxin. Moreover, the ability of RNF126 to promote frataxin ubiquitination in vitro, without additional cofactors, suggests that RNF126 can directly recognize and interact with frataxin precursor. However, we cannot rule out the possibility that in vivo RNF126 may be part of a multi-protein complex and frataxin recognition may require additional partners.

RNF126 has been implicated in a number of physiological and pathological processes, such as cancer progression, membrane receptor trafficking, and immune response modulation.

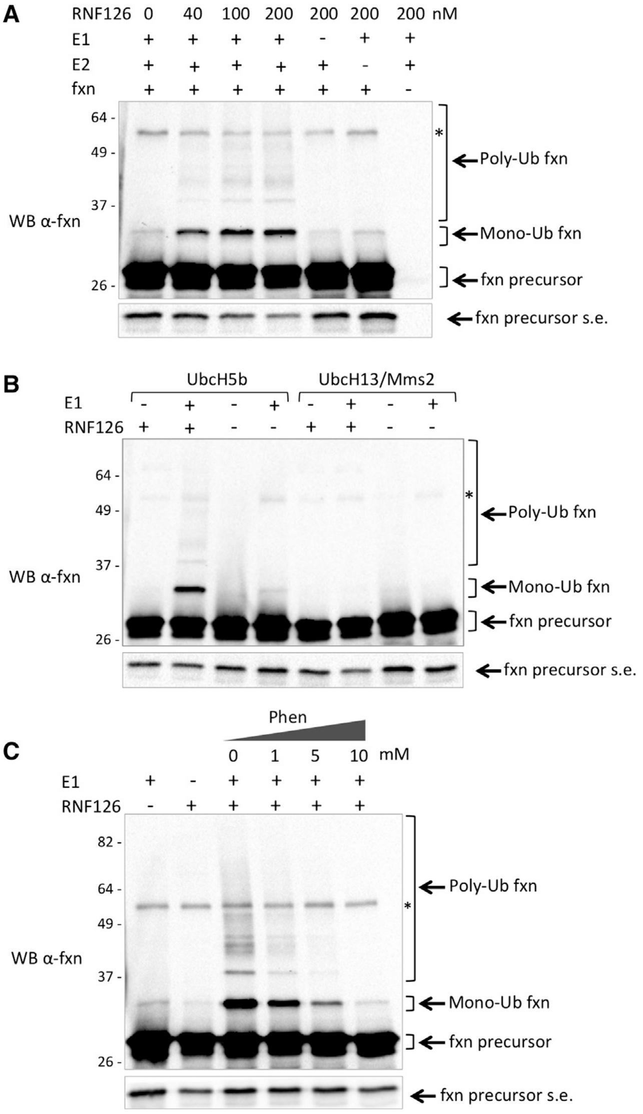

Figure 5. In Vitro Ubiquitination Assay

(A) In vitro ubiquitination assay was carried out with purified recombinant E1, UbcH5b as the E2 ubiquitin-conjugating enzyme, and different doses of GST-RNF126 as the E3 ubiquitin ligase, together with Ub, ATP, and recombinant frataxin precursor as a substrate. The reaction mixture was incubated for 60 min at 30°C. Reaction was stopped by the addition of 4× sample buffer. Proteins were separated on SDS-PAGE and analyzed by a WB with anti-frataxin antibody.

(B) Ubiquitination assay was carried out with the indicated E2 ubiquitin-conjugating enzymes and 200 nM RNF126. Proteins were analyzed as in (A).

(C) Ubiquitination assay was carried out as in (B), in the presence of the indicated concentration of 1,10-phenanthroline. Proteins were analyzed as in (A). Frataxin precursor, monoubiquitinated frataxin, and polyubiquitinated frataxin are indicated by arrows. Fxn precursor s.e. indicates a short exposure of the blot to appreciate precursor levels. *Non-specific bands.

RNF126 was shown to ubiquitinate and promote the degradation of the tumor suppressor p21 (Zhi et al., 2013) and to enhance the Akt signaling pathway (Wang et al., 2016). Moreover, it was shown to regulate the endosomal sorting of the epidermal growth factor receptor (EGFR) (Smith et al., 2013) and cation-independent mannose 6-phosphate receptor (CI-MPR) (Smith and McGlade, 2014) and to induce ubiquitination of the activation-induced cytidine deaminase (AID), an enzyme involved in the process of antibody diversification in the immune system (Delker et al., 2013). These data implicate the function of RNF126 in the regulation of an array of cellular responses and in different subcellular compartments. A paper indicates a role for RNF126 in promoting resistance to anoikis by degrading pyruvate dehydrogenase kinase (PDK) and allowing increased flux toward tricarboxylic acid (TCA) cycle, thus conferring metastatic ability on cancer cells (Yoshino et al., 2016). RNF126 has been identified as the E3 ligase in the Bag6 complex (Krysztofinska et al., 2016; Rodrigo-Brenni et al., 2014), a multiprotein complex implicated in the protein quality-control process and in particular dedicated to the degradation of mislocalized proteins (Binici and Koch, 2014; Leznicki et al., 2013). Protein translocation into organelles requires tight quality control to avoid mislocalization of proteins to the cytosol. Any factors affecting the efficiency of the translocation and import machinery may lead to unwanted accumulation of precursor proteins in the cytosolic compartment. In this perspective, proteins that are synthesized in their precursor form in the cytosol before their import into organelles, such as mitochondria, maybe considered “transiently mislocalized” proteins. It is interesting to speculate that frataxin precursor recognition by RNF126 may be part of such a dedicated quality-control mechanism.

Given the complexity of the different forms of frataxin and its maturation process (Condò et al., 2007), it is reasonable to consider the possibility that precursor accumulation may not result in increased mature generation. This may be the case when drugs are used that alter the efficiency of import and maturation machinery (Nabhan et al., 2015). However, here we show that in 293 Flp-In cells stably expressing frataxin$^{1–210}$, RNF126 knockdown results in an increase in all forms of frataxin in 48 hr, indicating that the increased precursor that is saved from degradation can be converted into intermediate and mature forms. Moreover, the accumulation of mature frataxin in cells derived from patients upon depletion of RNF126 suggests that this control mechanism is active in conditions of reduced frataxin expression and suggests the relevance of therapeutic approaches aimed at interfering with this process. Any therapeutic attempt to increase the amount of frataxin should take into account the existence of such a control mechanism. A combined therapy aimed at increasing frataxin levels, either by promoting its gene transcription or through gene therapy or protein replacement, and simultaneously interfering with its RNF126-mediated degradation could be envisioned.

Structural insight into the recognition mode between frataxin and RNF126 will provide useful information to drive the design of small molecules that either inhibit the catalytic activity of RNF126 or prevent its interaction with frataxin. In particular, given the biological importance of the quality-control process that involves RNF126, inhibiting the specific interaction between frataxin and RNF126, without perturbing its catalytic activity, may be a more desirable approach.

OPEN
ACCESS
CellPress

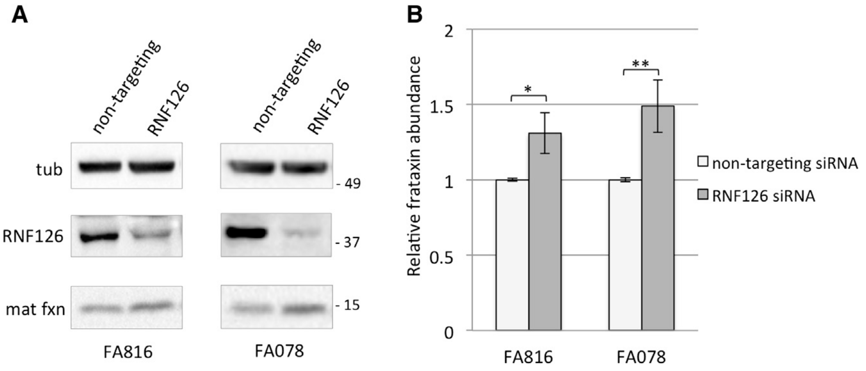

Figure 6. Silencing of RNF126 E3 Ligase Promotes Frataxin Accumulation in Cells Derived from FRDA Patients

(A) Fibroblasts derived from two FRDA patients (FA816 and FA078) were transfected with the indicated siRNA. Cell extracts were collected 48 hr post-transfection and analyzed by WB with anti-frataxin antibody, anti-RNF126, and anti-tubulin. Tub, tubulin; mat fxn, mature frataxin.

(B) Graph representing relative mature frataxin abundance as quantitated by densitometric analysis of the blots in (A) and normalized with tubulin levels.

Data represent the mean ± 1 SEM from four independent experiments. The p values were calculated with Student's t test and were statistically significant (*$p < 0.05$; **$p < 0.01$) compared to non-targeting siRNA-transfected cells.

EXPERIMENTAL PROCEDURES

E3 Ubiquitin Ligase siRNA Library Screening

The Human On-TargetPlus Reverse Transfection Format (RTF) Ubiquitin Conjugation siRNA library was purchased from Dharmacon and consisted of nine distinct 96-well plates supplied in six replicates. For the screening, HEK293 cells transiently transfected with pCMV-fxn-ProLabel were reverse transfected with 50 nM of the different siRNA pools prearrayed into individual wells, according to the manufacturer's instructions. Each pool consisted of four siRNA per gene. Cyclophilin B and non-targeting siRNA were used as positive and negative controls, respectively, and Alexa Fluor siRNA was used as transfection efficiency control. MG132-treated cells were included in each plate and considered internal positive controls. Briefly, Dharmafect 1 transfection reagent diluted in Dharmafect Cell Culture Reagent was added to On-TargetPlus RTF plates in a total volume of 25 μL per well to rehydrate and complex the prearrayed siRNA (step 1). After a short incubation of 30 min at room temperature, HEK293 cells expressing pCMV-fxn-ProLabel plasmid were plated at 25,000 cells per well for the reverse transfection (step 2). After 48 hr, the ProLabel enzyme fragment complementation assay was performed (step 3) using the ProLabel Detection Kit (Clontech Laboratories), according to the manufacturer's instructions. The chemiluminescent signal was monitored over 180 min.

The siRNA screening results were analyzed according to recommendations (Birmingham et al., 2009). This included data triage through plate visualization as heatmaps and hit rate calculation, which revealed no significant inhomogeneities or edge effects. Inter-plate normalization was performed through the robust *Z* score method to render data comparable across plates while reducing the effect of outliers. To assess assay quality, we calculated the Z' factor by pooling all high-value controls and low-value controls and obtained $Z' = 0.40$, indicating an acceptable to good assay. Successively, hits were identified on each plate through the median ± k median absolute deviation method, again to reduce the influence in outliers (Chung et al., 2008). Specifically, siRNAs giving a luminescence signal that was greater than three median absolute deviations above the plate median in at least four of six replicates were considered positive hits.

Cell Culture and Transfection Conditions

HEK293 cells were grown at 37°C and 5% CO_2 in DMEM supplemented with 10% fetal bovine serum (FBS), 100 U/mg penicillin/streptomycin, and 2 mM L-glutamine. Transfections were performed using the Lipofectamine 2000 method (Invitrogen), according to the manufacturer's instructions. Where indicated, the day after transfection, HEK293 cells were treated for 24 hr with 10 μM proteasome inhibitor MG132 (Sigma-Aldrich) and 50 ng/mL deubiquitinating enzyme (DUB) inhibitor Ub-aldehyde.

HEK293 Flp-In cells (Invitrogen) are HEK293 variants allowing the stable and isogenic integration and expression of a transfected gene. The HEK293 clone stably expressing frataxin$^{1–210}$ was previously described (Condò et al., 2007). Cells were maintained in DMEM supplemented with 10% FBS, 100 U/mg of the antibiotics penicillin/streptomycin, and 2 mM L-glutamine. Cells were transfected with a 50 nM siRNA pool containing four siRNAs for each gene (Table S1), or 50 nM individual siRNA where indicated (Table S2), using Dharmafect transfection reagents, according to the manufacturer's instruction.

FA816 fibroblasts from a clinically affected Friedreich ataxia patient, homozygous for the GAA expansion in the FRDA gene, with alleles containing 380 and 330 repeats, and FA078 fibroblasts from a second clinically affected patient, with alleles containing 541 and 420 repeats, were obtained from the National Institute of General Medical Sciences (NIGMS) Human Genetic Cell Repository, Coriell Institute for Medical Research. Cells were cultured in DMEM supplemented with 15% FBS, 100 U/mg penicillin/streptomycin, and 2 mM L-glutamine. Cells were transfected with a 25 nM siRNA pool containing four siRNAs for each gene (Table S1), or 25 nM individual siRNA where indicated (Table S2), using Dharmafect transfection reagents, according to the manufacturer's instructions. siGLOgreen was used as an indicator of relative delivery efficiency during silencing experiments.

cDNA Expression Constructs

The pIRES2-fxn$^{1–210}$ construct contains human frataxin cDNA cloned into pIRES2-EGFP (Clontech) bicistronic expression vector and was previously generated in our laboratory (Condò et al., 2006). pCMV-fxn-ProLabel construct was generated by subcloning PCR using the primers 5′-TAATAC GACTCACTATAGGG-3′ (T7) and 5′-GGGGATCCAGCATCTTTTCCGGAATA GGC-3′ (BamHI, no stop) to amplify frataxin cDNA from the plasmid pIRES2-fxn$^{1–210}$. Frataxin cDNA was then inserted between *Hind*III and BamHI restriction sites of the expression vector pCMV-ProLabel-C1, provided by Dr. Claudio Joazeiro (The Scripps Research Institute). The HA-Ub construct was generated by M. Treier in Dirk Bohmann's lab (Treier et al., 1994). The plasmid vectors encoding for RNF126 (Myc-FLAG-tagged)-human ring finger protein 126 (pCMV6-RNF126), SYVN1 (Myc-FLAG-tagged)-human synovial apoptosis inhibitor 1 (pCMV6-SYVN1), and FBXO44 (Myc-FLAG-tagged)-human F-box protein 44 (pCMV6-FBXO44) were obtained from OriGene. The RNF126$^{C229/232A}$ construct was generated using the Quick-Change site-directed mutagenesis kit (Agilent Technologies) with specific primers using pCMV6-RNF126 as a template. The sequences of the DNA constructs generated by PCR were systematically verified by DNA sequencing.

Cell Lysates, Western Blot Analysis, and Antibodies

Total cell extracts were prepared in lysis buffer (50 mM Tris-HCl [pH 7.5], 150 mM NaCl, 1% Igepal CA-630, 5 mM EDTA, 5 mM EGTA) supplemented with complete protease inhibitor cocktail (Roche Diagnostics). For in vivo detection of ubiquitin conjugates, 10 μM MG132 (Sigma-Aldrich), 50 ng/mL Ub-aldehyde (Biomol), and 2 mM N-ethylmaleimide (NEM, Sigma-Aldrich) were added to the lysis buffer. Lysates were clarified by centrifugation, and supernatants were mixed with 4× Laemmli sample buffer (50 mM Tris [pH 6.8], 2% SDS, 10% glycerol, 0.0025% bromophenol blue, 5% β-mercaptoethanol)

and boiled for 5 min. Then, 50 μg of protein extract were resolved by precast 10% or 12% SDS-PAGE gels (Bio-Rad) and transferred to 0.2 μM nitrocellulose membrane (Trans-Blot Turbo Transfer pack, Bio-Rad). Membranes were blocked with 5% non-fat dry milk in 0.1% Tween 20 PBS and incubated with the indicated primary and secondary antibodies. The immunoreactive bands were detected by enhanced chemiluminescence (ECL, GE Healthcare) and imaged with a ChemiDoc XRS system (Bio-Rad). Densitometric analysis was performed using ImageLab 4.1 software (Bio-Rad). To analyze the western blot (WB) densitometric results, a Student's t test was applied. All values are expressed as means ± 1 SEM.

The following antibodies were used for immunoprecipitation and western blot analysis: monoclonal antibody (mAb) anti-frataxin (clone 18A5DB1, Abcam) and polyclonal antibody (pAb) anti-frataxin (Abcam), mAb anti-α-tubulin (clone DM1A, Sigma-Aldrich), mAb anti-HA (clone HA-7, Sigma-Aldrich), mAb anti-RNF126 (Santa Cruz Biotechnology), rabbit monoclonal antibody (RabmAb) anti-Hsp60 (Abcam), RabmAb anti-RNF115 (Abcam), mAb anti-FLAG (clone M2, Sigma-Aldrich), and secondary antibody horseradish peroxidase (HRP)-conjugated goat anti-mouse (Thermo Fisher Scientific).

Immunoprecipitation

Total cell extracts were prepared in ice-cold modified immunoprecipitate (IP) buffer (25 mM Tris-HCl [pH 7.5], 125 mM NaCl, 1% glycerol, 1 mM $MgCl_2$, 0.5% Triton, 0.5% Igepal CA-630, 0.5% sodium deoxycholate) supplemented with complete protease inhibitor cocktail (Roche), 5 mM NEM, 1 mM sodium orthovanadate (NaV), and 5 mM sodium fluoride (NaF) to inhibit phosphatases. For immunoprecipitation experiments, 2 mg of whole-cell lysates were incubated for 1–2 hr at 4°C with the appropriated amount of mouse monoclonal anti-frataxin (Abcam) or mouse monoclonal anti-RNF126 (Santa Cruz) antibodies per sample. The immunocomplexes were then absorbed for 1–2 hr at 4°C on prewashed protein glutathione Sepharose beads (GE Healthcare) and finally washed three times in wash buffer (25 mM Tris-HCl [pH 7.5], 125 mM NaCl, 1% glycerol, 1 mM $MgCl_2$, 0.5% Igepal CA-630) supplemented with complete protease inhibitor cocktail, 5 mM NEM, 1 mM NaV, and 5 mM NaF. After washing, immunocomplexes were resuspended in 2× Laemmli sample buffer, boiled for 5 min, resolved by SDS-PAGE, and analyzed by WB with rabbit polyclonal anti-frataxin (Abcam) or mouse monoclonal anti-RNF126 (Santa Cruz). Densitometric analyzes were performed using ImageLab software.

Ubiquitination Assay

In vivo ubiquitination assay was performed using HEK293 cells, transiently cotransfected with cDNAs encoding human frataxin$^{1–210}$ (pIRES2-fxn), HA-tagged ubiquitin (HA-Ub), and RNF126 (pCMV6-RNF126) or RNF126 catalytically inactive mutant (RNF126$^{C229/232A}$). Before collecting, cells were treated with 10 μM MG132 (Sigma-Aldrich) and 50 ng/mL Ub-aldehyde (Biomol). To immunoprecipitate HA-frataxin, cells were lysed in lysis buffer (50 mM Tris-HCl [pH 7.5], 150 mM NaCl, 1% Igepal CA-630, 5 mM EDTA, 5 mM EGTA) in the presence of 10 μM MG132, 50 ng/mL Ub-aldehyde, and 2 mM NEM (Sigma-Aldrich). Mono- and polyubiquitinated frataxin species were detected by WB using mouse monoclonal anti-frataxin antibody (Abcam).

The in vitro ubiquitination assay was performed in a reaction mixture containing 100 nM of bacterially purified human recombinant His-tagged E1, 1.4 μM of a human recombinant untagged E2 (UbcH5b or UbcH13/Mms2) and 40–200 nM of human purified recombinant glutathione S-transferase (GST)-RNF126 (E3) in ubiquitination buffer (25 mM Tris-HCl [pH 8.0], 100 mM NaCl, 1 mM DTT, 2.5 mM ATP, 4 mM $MgCl_2$, 30 nM Ub) in a final volume of 20 μL. The human recombinant frataxin$^{1–210}$ protein expressed in *E. coli* was purchased from GenScript. Recombinant ubiquitin was purchased from Enzo Life Sciences. Human recombinant GST-RNF126 protein, purified using an in vitro wheat germ expression system, was purchased from Abnova. 1,10-Phenanthroline monohydrochloride monohydrate (1,10-phenanthroline) was purchased from Sigma-Aldrich and, where indicated, was preincubated with RNF126 for 5 min before starting the assay. After incubation for 60 min at 30°C, the reactions were terminated by adding 4× Laemmli sample buffer, loaded on a 10% Mini-Protean TGX Stain-Free Gel (Bio-Rad), and followed by WB with monoclonal anti-frataxin antibody (Abcam).

Real-Time qPCR

Total RNA isolated from 293 Flp-In cell line stably expressing frataxin1-210 and FRDA fibroblasts was extracted using TRIzol reagent (Thermo Fisher), and then cDNA was prepared using the SuperScript VILO cDNA synthesis kit (Thermo Fisher). Levels of human FXN and RNF126 mRNA expression were assessed by real-time qPCR using the StepOne Plus Instrument, normalized with the control genes' expression. The assays were performed using the following TaqMan assay: FTX, Hs00175940_m1; RNF126, Hs00250449_m1; GAPDH, Hs99999905_m1; and GUSB: Hs99999908_m1. GUSB and GAPDH were used as control housekeeping genes. Real-time qPCR analysis was carried out using the 2(-Delta Delta C(T)) method.

SUPPLEMENTAL INFORMATION

Supplemental Information includes six figures and two tables and can be found with this article online at http://dx.doi.org/10.1016/j.celrep.2017.01.079.

AUTHOR CONTRIBUTIONS

Conceptualization, M.B., S.F., I.C., F.M., R.T., and A.R.; Methodology, M.B., I.C., N.T., G. Arcuri, and A.R.; Formal Analysis, G. Alfedi, N.T., and A.R.; Investigation, M.B., S.F., G. Alfedi, D.S., D.S.M., and G. Arcuri; Writing – Original Draft, A.R.; Writing – Review & Editing, M.B., I.C., G. Alfedi, N.T., R.T., and A.R.; Funding Acquisition, R.T.; Supervision, R.T. and A.R.

ACKNOWLEDGMENTS

We are grateful to Dr. Claudio Joazeiro (The Scripps Research Institute) for kindly providing the plasmid pCMV-ProLabel and assisting in the initial setting of the screening and to Dr. Angelo Peschiaroli (Institute of Cell Biology and Neurobiology [IBCN], CNR) for helpful suggestions. This work was supported by grants from the European Research Council (Advanced Grant #293699, FAST) and Telethon, Italy (GGP11102). A.R. is the Scientific Director of Fratagene Therapeutics srl, and R.T. is the Founder of Fratagene Therapeutics srl.

Received: August 10, 2016
Revised: December 14, 2016
Accepted: January 29, 2017
Published: February 21, 2017

REFERENCES

Al-Mahdawi, S., Pinto, R.M., Ismail, O., Varshney, D., Lymperi, S., Sandi, C., Trabzuni, D., and Pook, M. (2008). The Friedreich ataxia GAA repeat expansion mutation induces comparable epigenetic changes in human and transgenic mouse brain and heart tissues. Hum. Mol. Genet. *17*, 735–746.

Alper, G., and Narayanan, V. (2003). Friedreich's ataxia. Pediatr. Neurol. *28*, 335–341.

Anzovino, A., Lane, D.J., Huang, M.L., and Richardson, D.R. (2014). Fixing frataxin: 'ironing out' the metabolic defect in Friedreich's ataxia. Br. J. Pharmacol. *171*, 2174–2190.

Berndsen, C.E., and Wolberger, C. (2014). New insights into ubiquitin E3 ligase mechanism. Nat. Struct. Mol. Biol. *21*, 301–307.

Binici, J., and Koch, J. (2014). BAG-6, a jack of all trades in health and disease. Cell. Mol. Life Sci. *71*, 1829–1837.

Birmingham, A., Selfors, L.M., Forster, T., Wrobel, D., Kennedy, C.J., Shanks, E., Santoyo-Lopez, J., Dunican, D.J., Long, A., Kelleher, D., et al. (2009). Statistical methods for analysis of high-throughput RNA interference screens. Nat. Methods *6*, 569–575.

Bulteau, A.L., O'Neill, H.A., Kennedy, M.C., Ikeda-Saito, M., Isaya, G., and Szweda, L.I. (2004). Frataxin acts as an iron chaperone protein to modulate mitochondrial aconitase activity. Science *305*, 242–245.

Campuzano, V., Montermini, L., Moltò, M.D., Pianese, L., Cossée, M., Cavalcanti, F., Monros, E., Rodius, F., Duclos, F., Monticelli, A., et al. (1996).

Friedreich's ataxia: autosomal recessive disease caused by an intronic GAA triplet repeat expansion. Science *271*, 1423–1427.

Campuzano, V., Montermini, L., Lutz, Y., Cova, L., Hindelang, C., Jiralerspong, S., Trottier, Y., Kish, S.J., Faucheux, B., Trouillas, P., et al. (1997). Frataxin is reduced in Friedreich ataxia patients and is associated with mitochondrial membranes. Hum. Mol. Genet. *6*, 1771–1780.

Chung, N., Zhang, X.D., Kreamer, A., Locco, L., Kuan, P.F., Bartz, S., Linsley, P.S., Ferrer, M., and Strulovici, B. (2008). Median absolute deviation to improve hit selection for genome-scale RNAi screens. J. Biomol. Screen. *13*, 149–158.

Chutake, Y.K., Costello, W.N., Lam, C., and Bidichandani, S.I. (2014a). Altered nucleosome positioning at the transcription start site and deficient transcriptional initiation in Friedreich ataxia. J. Biol. Chem. *289*, 15194–15202.

Chutake, Y.K., Lam, C., Costello, W.N., Anderson, M., and Bidichandani, S.I. (2014b). Epigenetic promoter silencing in Friedreich ataxia is dependent on repeat length. Ann. Neurol. *76*, 522–528.

Ciechanover, A. (2013). Intracellular protein degradation: from a vague idea through the lysosome and the ubiquitin-proteasome system and onto human diseases and drug targeting. Bioorg. Med. Chem. *21*, 3400–3410.

Cnop, M., Mulder, H., and Igoillo-Esteve, M. (2013). Diabetes in Friedreich ataxia. J. Neurochem. *126* (*Suppl 1*), 94–102.

Cohen, P., and Tcherpakov, M. (2010). Will the ubiquitin system furnish as many drug targets as protein kinases? Cell *143*, 686–693.

Collins, A. (2013). Clinical neurogenetics: Friedreich ataxia. Neurol. Clin. *31*, 1095–1120.

Condò, I., Ventura, N., Malisan, F., Tomassini, B., and Testi, R. (2006). A pool of extramitochondrial frataxin that promotes cell survival. J. Biol. Chem. *281*, 16750–16756.

Condò, I., Ventura, N., Malisan, F., Rufini, A., Tomassini, B., and Testi, R. (2007). In vivo maturation of human frataxin. Hum. Mol. Genet. *16*, 1534–1540.

Condò, I., Malisan, F., Guccini, I., Serio, D., Rufini, A., and Testi, R. (2010). Molecular control of the cytosolic aconitase/IRP1 switch by extramitochondrial frataxin. Hum. Mol. Genet. *19*, 1221–1229.

Delatycki, M.B., Williamson, R., and Forrest, S.M. (2000). Friedreich ataxia: an overview. J. Med. Genet. *37*, 1–8.

Delker, R.K., Zhou, Y., Strikoudis, A., Stebbins, C.E., and Papavasiliou, F.N. (2013). Solubility-based genetic screen identifies RING finger protein 126 as an E3 ligase for activation-induced cytidine deaminase. Proc. Natl. Acad. Sci. USA *110*, 1029–1034.

Deshaies, R.J., and Joazeiro, C.A. (2009). RING domain E3 ubiquitin ligases. Annu. Rev. Biochem. *78*, 399–434.

Eglen, R.M. (2002). Enzyme fragment complementation: a flexible high throughput screening assay technology. Assay Drug Dev. Technol. *1*, 97–104.

Evans-Galea, M.V., Pébay, A., Dottori, M., Corben, L.A., Ong, S.H., Lockhart, P.J., and Delatycki, M.B. (2014). Cell and gene therapy for Friedreich ataxia: progress to date. Hum. Gene Ther. *25*, 684–693.

Grabczyk, E., Mancuso, M., and Sammarco, M.C. (2007). A persistent RNA.DNA hybrid formed by transcription of the Friedreich ataxia triplet repeat in live bacteria, and by T7 RNAP in vitro. Nucleic Acids Res. *35*, 5351–5359.

Groh, M., Lufino, M.M., Wade-Martins, R., and Gromak, N. (2014). R-loops associated with triplet repeat expansions promote gene silencing in Friedreich ataxia and fragile X syndrome. PLoS Genet. *10*, e1004318.

Herman, D., Jenssen, K., Burnett, R., Soragni, E., Perlman, S.L., and Gottesfeld, J.M. (2006). Histone deacetylase inhibitors reverse gene silencing in Friedreich's ataxia. Nat. Chem. Biol. *2*, 551–558.

Igoillo-Esteve, M., Gurgul-Convey, E., Hu, A., Romagueira Bichara Dos Santos, L., Abdulkarim, B., Chintawar, S., Marselli, L., Marchetti, P., Jonas, J.C., Eizirik, D.L., et al. (2015). Unveiling a common mechanism of apoptosis in β-cells and neurons in Friedreich's ataxia. Hum. Mol. Genet. *24*, 2274–2286.

Kim, E., Napierala, M., and Dent, S.Y. (2011). Hyperexpansion of GAA repeats affects post-initiation steps of FXN transcription in Friedreich's ataxia. Nucleic Acids Res. *39*, 8366–8377.

Koeppen, A.H., and Mazurkiewicz, J.E. (2013). Friedreich ataxia: neuropathology revised. J. Neuropathol. Exp. Neurol. *72*, 78–90.

Koeppen, A.H., Ramirez, R.L., Becker, A.B., Bjork, S.T., Levi, S., Santambrogio, P., Parsons, P.J., Kruger, P.C., Yang, K.X., Feustel, P.J., and Mazurkiewicz, J.E. (2015). The pathogenesis of cardiomyopathy in Friedreich ataxia. PLoS ONE *10*, e0116396.

Krysztofinska, E.M., Martínez-Lumbreras, S., Thapaliya, A., Evans, N.J., High, S., and Isaacson, R.L. (2016). Structural and functional insights into the E3 ligase, RNF126. Sci. Rep. *6*, 26433.

Lane, D.J., Huang, M.L., Ting, S., Sivagurunathan, S., and Richardson, D.R. (2013). Biochemistry of cardiomyopathy in the mitochondrial disease Friedreich's ataxia. Biochem. J. *453*, 321–336.

Leznicki, P., Roebuck, Q.P., Wunderley, L., Clancy, A., Krysztofinska, E.M., Isaacson, R.L., Warwicker, J., Schwappach, B., and High, S. (2013). The association of BAG6 with SGTA and tail-anchored proteins. PLoS ONE *8*, e59590.

Li, Y., Lu, Y., Polak, U., Lin, K., Shen, J., Farmer, J., Seyer, L., Bhalla, A.D., Rozwadowska, N., Lynch, D.R., et al. (2015). Expanded GAA repeats impede transcription elongation through the FXN gene and induce transcriptional silencing that is restricted to the FXN locus. Hum. Mol. Genet. *24*, 6932–6943.

Lill, J.R., and Wertz, I.E. (2014). Toward understanding ubiquitin-modifying enzymes: from pharmacological targeting to proteomics. Trends Pharmacol. Sci. *35*, 187–207.

Lynch, D.R., Deutsch, E.C., Wilson, R.B., and Tennekoon, G. (2012). Unanswered questions in Friedreich ataxia. J. Child Neurol. *27*, 1223–1229.

Martelli, A., and Puccio, H. (2014). Dysregulation of cellular iron metabolism in Friedreich ataxia: from primary iron-sulfur cluster deficit to mitochondrial iron accumulation. Front. Pharmacol. *5*, 130.

Metzger, M.B., Pruneda, J.N., Klevit, R.E., and Weissman, A.M. (2014). RING-type E3 ligases: master manipulators of E2 ubiquitin-conjugating enzymes and ubiquitination. Biochim. Biophys. Acta *1843*, 47–60.

Nabhan, J.F., Gooch, R.L., Piatnitski Chekler, E.L., Pierce, B., and Bulawa, C.E. (2015). Perturbation of cellular proteostasis networks identifies pathways that modulate precursor and intermediate but not mature levels of frataxin. Sci. Rep. *5*, 18251.

Nagy, V., and Dikic, I. (2010). Ubiquitin ligase complexes: from substrate selectivity to conjugational specificity. Biol. Chem. *391*, 163–169.

Pastore, A., and Puccio, H. (2013). Frataxin: a protein in search for a function. J. Neurochem. *126* (*Suppl 1*), 43–52.

Popovic, D., Vucic, D., and Dikic, I. (2014). Ubiquitination in disease pathogenesis and treatment. Nat. Med. *20*, 1242–1253.

Richardson, D.R., Huang, M.L., Whitnall, M., Becker, E.M., Ponka, P., and Suryo Rahmanto, Y. (2010). The ins and outs of mitochondrial iron-loading: the metabolic defect in Friedreich's ataxia. J. Mol. Med. (Berl.) *88*, 323–329.

Rodrigo-Brenni, M.C., Gutierrez, E., and Hegde, R.S. (2014). Cytosolic quality control of mislocalized proteins requires RNF126 recruitment to Bag6. Mol. Cell *55*, 227–237.

Rouault, T.A., and Tong, W.H. (2008). Iron-sulfur cluster biogenesis and human disease. Trends Genet. *24*, 398–407.

Rufini, A., Fortuni, S., Arcuri, G., Condò, I., Serio, D., Incani, O., Malisan, F., Ventura, N., and Testi, R. (2011). Preventing the ubiquitin-proteasome-dependent degradation of frataxin, the protein defective in Friedreich's ataxia. Hum. Mol. Genet. *20*, 1253–1261.

Rufini, A., Cavallo, F., Condò, I., Fortuni, S., De Martino, G., Incani, O., Di Venere, A., Benini, M., Massaro, D.S., Arcuri, G., et al. (2015). Highly specific ubiquitin-competing molecules effectively promote frataxin accumulation and partially rescue the aconitase defect in Friedreich ataxia cells. Neurobiol. Dis. *75*, 91–99.

Schmidt, M., and Finley, D. (2014). Regulation of proteasome activity in health and disease. Biochim. Biophys. Acta *1843*, 13–25.

Smith, C.J., and McGlade, C.J. (2014). The ubiquitin ligase RNF126 regulates the retrograde sorting of the cation-independent mannose 6-phosphate receptor. Exp. Cell Res. *320*, 219–232.

Smith, C.J., Berry, D.M., and McGlade, C.J. (2013). The E3 ubiquitin ligases RNF126 and Rabring7 regulate endosomal sorting of the epidermal growth factor receptor. J. Cell Sci. *126*, 1366–1380.

Soragni, E., Miao, W., Iudicello, M., Jacoby, D., De Mercanti, S., Clerico, M., Longo, F., Piga, A., Ku, S., Campau, E., et al. (2014). Epigenetic therapy for Friedreich ataxia. Ann. Neurol. *76*, 489–508.

Strawser, C.J., Schadt, K.A., and Lynch, D.R. (2014). Therapeutic approaches for the treatment of Friedreich's ataxia. Expert Rev. Neurother. *14*, 949–957.

Treier, M., Staszewski, L.M., and Bohmann, D. (1994). Ubiquitin-dependent c-Jun degradation in vivo is mediated by the delta domain. Cell *78*, 787–798.

Tsai, C.L., and Barondeau, D.P. (2010). Human frataxin is an allosteric switch that activates the Fe-S cluster biosynthetic complex. Biochemistry *49*, 9132–9139.

Vaubel, R.A., and Isaya, G. (2013). Iron-sulfur cluster synthesis, iron homeostasis and oxidative stress in Friedreich ataxia. Mol. Cell. Neurosci. *55*, 50–61.

Vetcher, A.A., Napierala, M., Iyer, R.R., Chastain, P.D., Griffith, J.D., and Wells, R.D. (2002). Sticky DNA, a long GAA.GAA.TTC triplex that is formed intramolecularly, in the sequence of intron 1 of the frataxin gene. J. Biol. Chem. *277*, 39217–39227.

Wang, L., Wang, X., Zhao, Y., Niu, W., Ma, G., Yin, W., and Shi, C. (2016). E3 ubiquitin ligase RNF126 regulates the progression of tongue cancer. Cancer Med. *5*, 2043–2047.

Weidemann, F., Störk, S., Liu, D., Hu, K., Herrmann, S., Ertl, G., and Niemann, M. (2013). Cardiomyopathy of Friedreich ataxia. J. Neurochem. *126* (*Suppl 1*), 88–93.

Wilson, R.B. (2012). Therapeutic developments in Friedreich ataxia. J. Child Neurol. *27*, 1212–1216.

Yoshino, S., Hara, T., Nakaoka, H.J., Kanamori, A., Murakami, Y., Seiki, M., and Sakamoto, T. (2016). The ERK signaling target RNF126 regulates anoikis resistance in cancer cells by changing the mitochondrial metabolic flux. Cell Discov. *2*, 16019.

Zhang, W., and Sidhu, S.S. (2014). Development of inhibitors in the ubiquitination cascade. FEBS Lett. *588*, 356–367.

Zhi, X., Zhao, D., Wang, Z., Zhou, Z., Wang, C., Chen, W., Liu, R., and Chen, C. (2013). E3 ubiquitin ligase RNF126 promotes cancer cell proliferation by targeting the tumor suppressor p21 for ubiquitin-mediated degradation. Cancer Res. *73*, 385–394.

Cell Reports, Volume *18*

Supplemental Information

E3 Ligase RNF126 Directly Ubiquitinates Frataxin, Promoting Its Degradation: Identification of a Potential Therapeutic Target for Friedreich Ataxia

Monica Benini, Silvia Fortuni, Ivano Condò, Giulia Alfedi, Florence Malisan, Nicola Toschi, Dario Serio, Damiano Sergio Massaro, Gaetano Arcuri, Roberto Testi, and Alessandra Rufini

SUPPLEMENTAL INFORMATION

Supplemental Figure 1 – Related to Figure 1

A

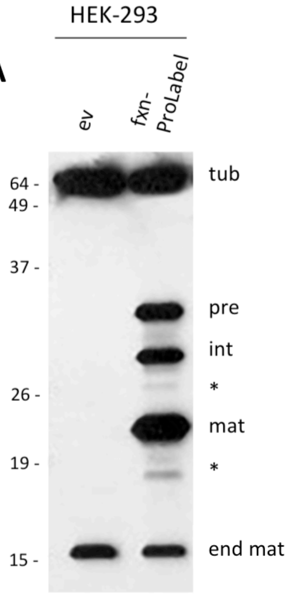

B

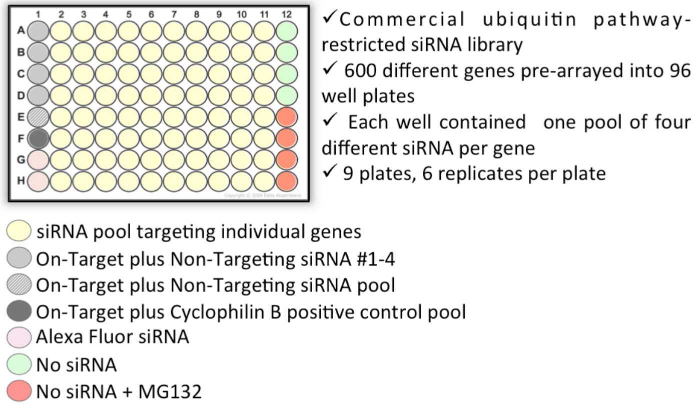

C

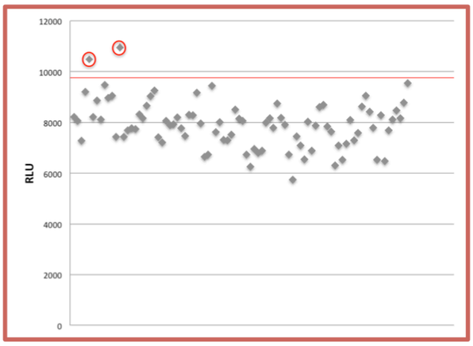

D

Positive replicates (out of 6)	Hit name	Median of NMD across replicates	Median deviation of NMD across replicates	Average FI across replicates	S.D. of FI across replicates
6	Cand1	7.54042	0.660279	1.61	0.16
6	RNF126	6.02534	0.330208	1.49	0.15
6	FBXO44	5.67972	0.549082	1.51	0.15
5	Syvn1	4.78579	1.03014	1.49	0.31
4	FBXW12	5.63934	0.993848	1.35	0.16
4	Cul1	3.87267	0.618967	1.29	0.12

Figure S1. Details of the screening set-up and hits identification.
A) Regular processing of the human frataxin precursor in the ProLabel fusion construct. HEK-293 cells were transiently transfected with empty vector (ev) or frataxin$^{1\text{-}210}$-ProLabel fusion construct (fxn-ProLabel) and analyzed by WB with anti-frataxin and anti-tubulin antibody. Pre: precursor; int: intermediate; mat: mature

frataxin forms expressed by the fxn-ProLabel construct; end mat: endogenous mature. The asterisks (*) denote non-specific bands.

B) Plate layout of Human ON-TARGETplus ubiquitin conjugation siRNA library (Dharmacon). Validated individual control siRNAs and pools are pre-dispensed into column 1 of each RTF library plate, providing a consistent baseline for screening and assay efficiency. Cells were transfected with Alexa Fluor siRNA in the indicated wells to control transfection efficiency. A column of no-siRNA transfected cells was included in each plate and cells treated with the proteasome inhibitor MG132 were included as positive control (column 12).

C) Representative results obtained in one of the screening plate. The graph shows the results obtained from the luminometer reading of one of the plates used for the screening. Each dot represents the RLU (Relative Luminescence Unit) measured in each individual well, corresponding to a determined siRNA pool. The red line indicates the arbitrary threshold that was set as the median of the luminescence measured in all the wells of the plate plus three median absolute deviations. Red dots highlight siRNA pools that were considered positive in this plate. Six replicates per plate were analyzed. siRNA pools that scored above the threshold in at least 4 out of 6 replicates were selected for further validation.

D) Table illustrating the primary hits identified from the siRNA screening. NMD: number of median deviations above the plate median (NMD > 3 was considered a hit on any single replicate). FI: fold increase over plate median (before normalization).

Supplemental Figure 2 – Related to Figure 2

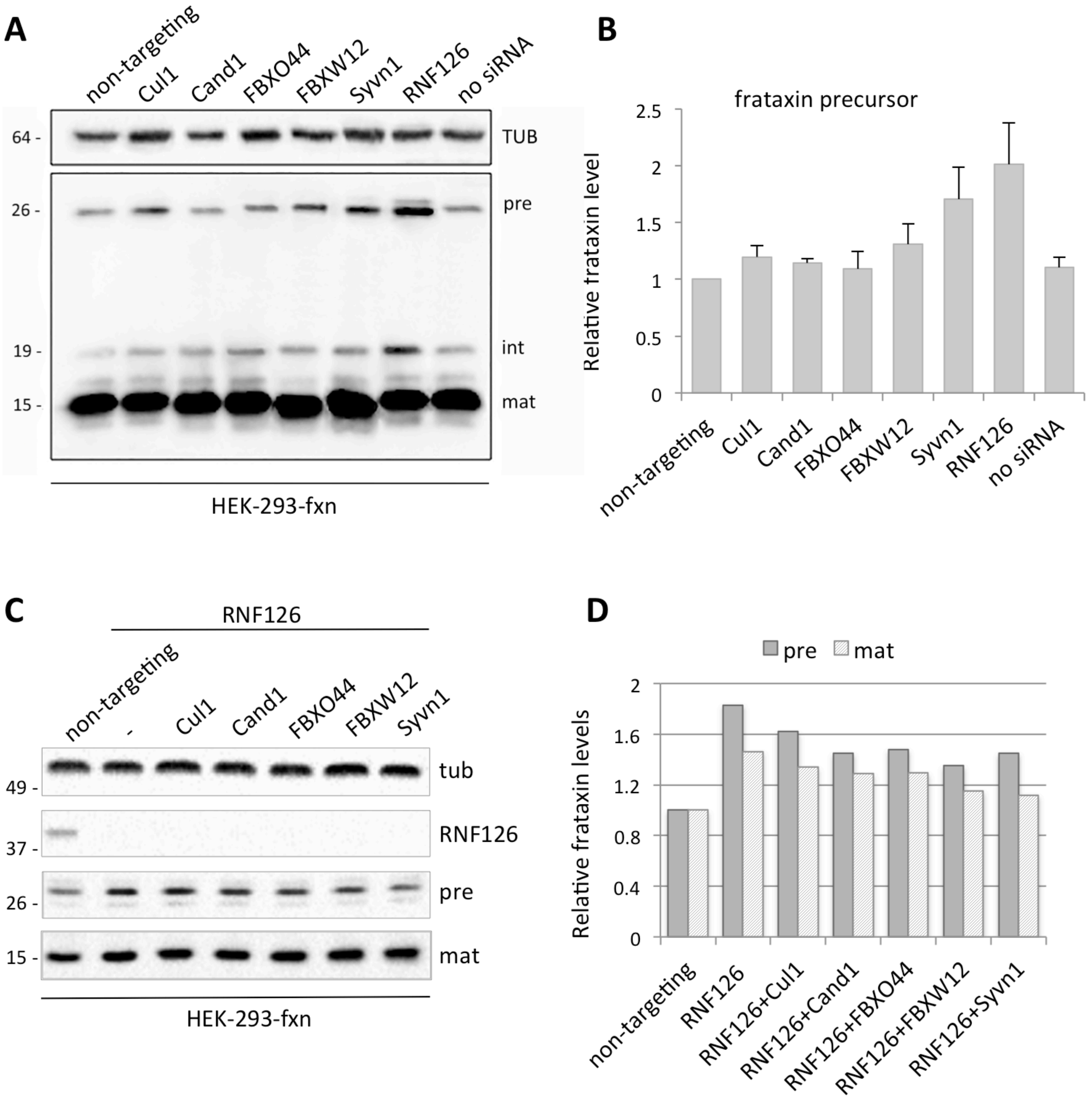

Figure S2. Primary hits validation.

A) siRNAs effect on frataxin levels. HEK-293 cell line stably expressing frataxin$^{1\text{-}210}$ were transfected with the indicated siRNAs. 48 hours after transfection, cells were collected and analyzed by SDS-PAGE and revealed by western blot with anti-frataxin antibody or anti-tubulin. pre: frataxin precursor; int: intermediate frataxin; mat: mature frataxin.

B) The graph represents the relative frataxin precursor levels, quantified as the densitometric ratio between frataxin precursor and tubulin for each lane. Data represent the mean ± 1 S.E.M. from five different independent experiments.

C) Simultaneous knock-down of RNF126 and the other hits identified in the screening does not promote further increase in frataxin levels. HEK-293 cells were transfected with the indicated combination of siRNA and analysed as in A. pre: frataxin

D) The graphs represent relative frataxin precursor (pre) and mature frataxin (mat) abundance as quantitated by densitometric analysis of the blots in C and normalized with tubulin levels. One representative experiment out of two performed with similar results is shown.

Supplemental Figure 3 – Related to Figures 2 and 6

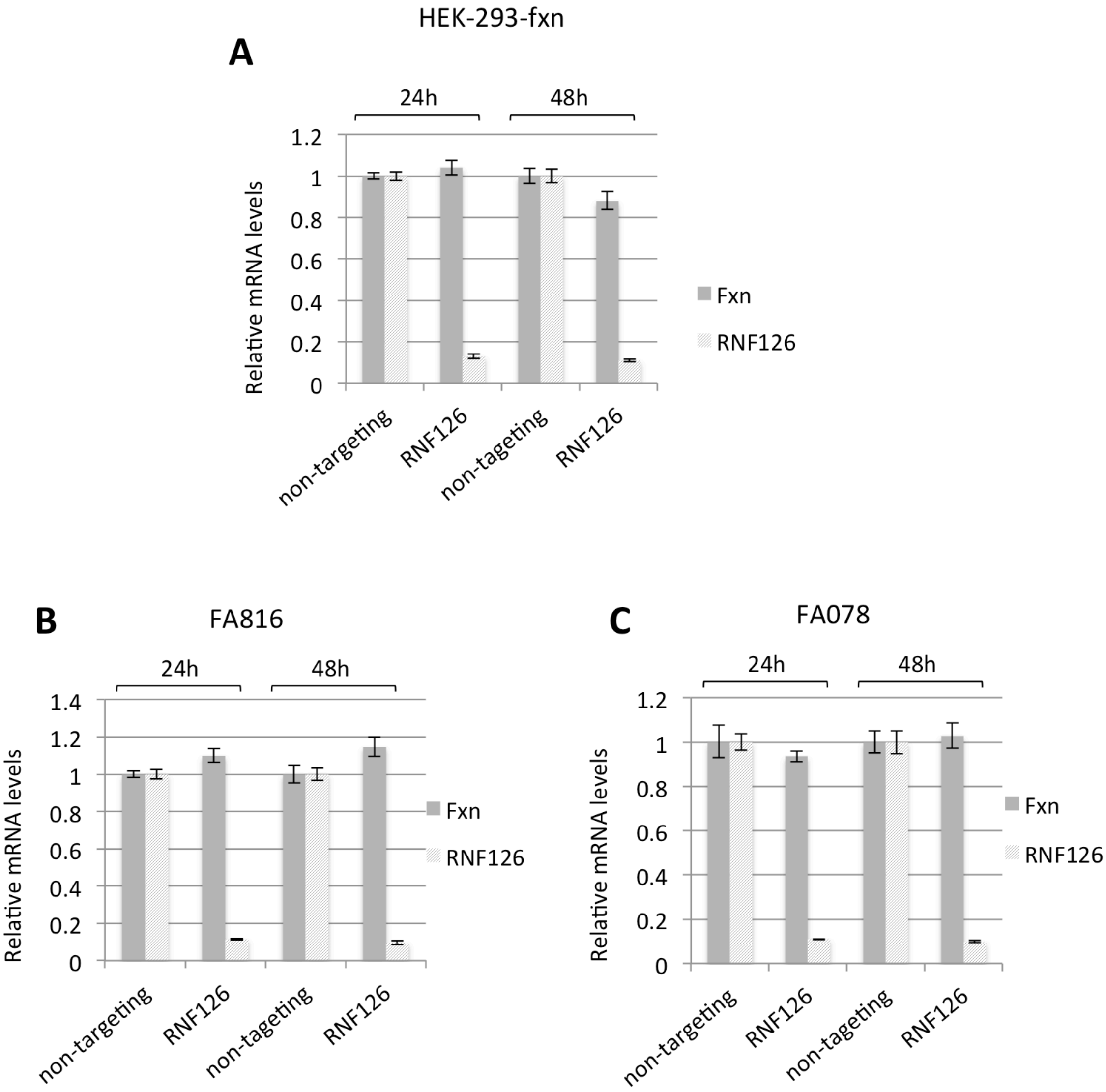

Fig. S3. Silencing of RNF126 E3 ligase does not alter frataxin mRNA levels.
A) 293 Flp-In cell line stably expressing frataxin$^{1-210}$ was transfected with 50 nM of either non-targeting siRNA or RNF126 siRNA, as indicated. Total RNA extracts were collected 24 and 48 hrs post-transfection and analyzed by Real Time PCR. One out of three experiments performed with similar results is shown in the panel. Error bars represent +/- 1SD of the $\Delta\Delta C_T$ value calculated on two technical replicates.
B-C) Fibroblasts derived from 2 different FRDA patients, FA816 (B) and FA078 (C) were transfected with 50 nM of either non-targeting siRNA or RNF126 siRNA, as indicated. Total RNA extracts were collected and analyzed as in A. Error bars represent +/- 1SD of the $\Delta\Delta C_T$ value calculated on two technical replicates.

Supplemental Figure 4 – Related to Figure 2

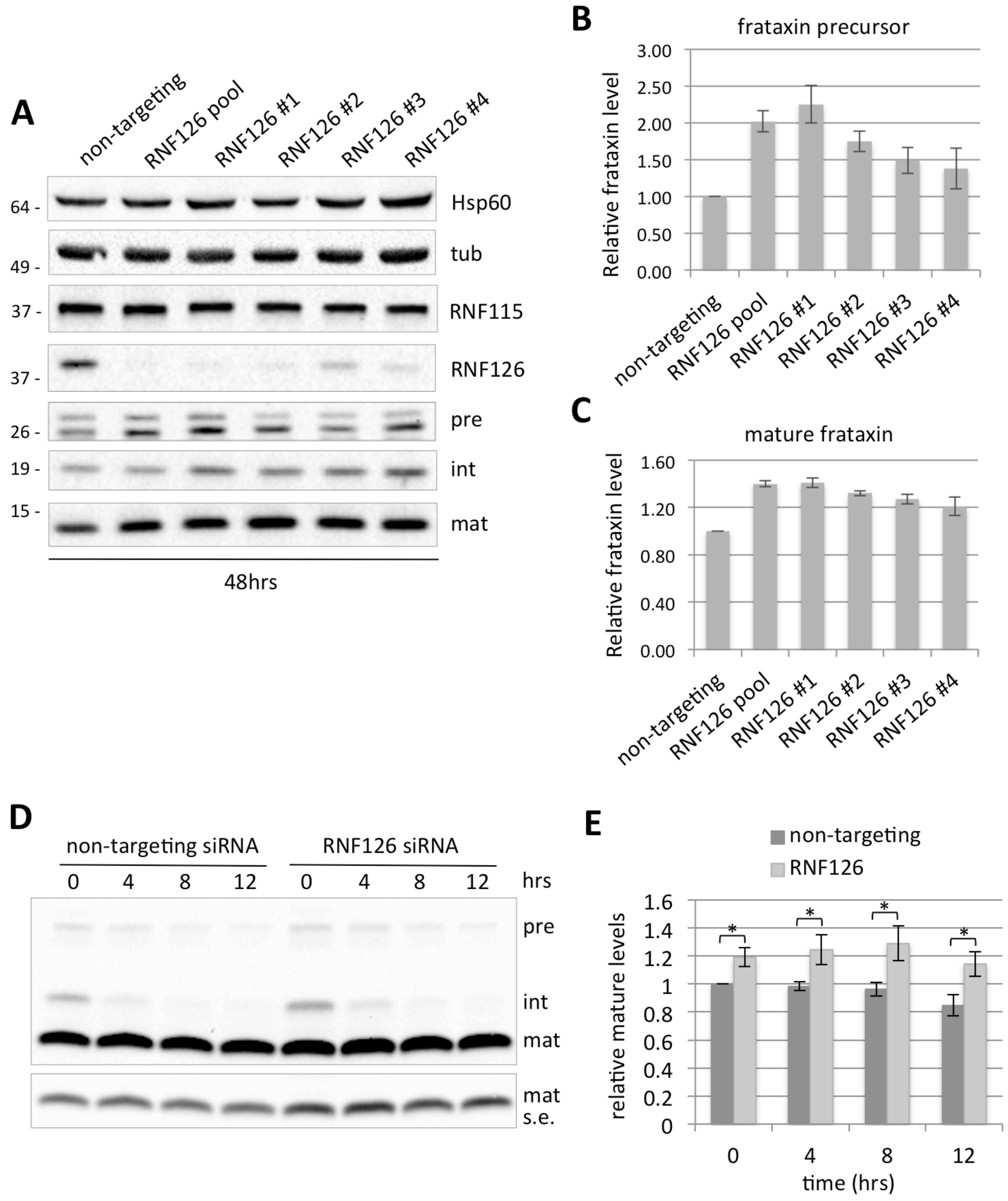

Figure S4. Silencing of RNF126 promotes frataxin accumulation and increases the frataxin precursor stability.

A) Results obtained in Fig 2 A-B using siRNA pools were confirmed with individual siRNA targeting RNF126. 293 Flp-In cell line stably expressing frataxin$^{1-210}$ was transfected with 50 nM of the indicated siRNA pool or 50 nM of the individual RNF126 siRNA. Cell extracts were collected 48 post-transfection and analyzed by WB with anti-frataxin, anti-RNF126, anti-RNF115 and anti HSP60 and anti-tubulin antibody. Pre: precursor; int: intermediate; mat: mature frataxin.

B-C) The graphs represent relative frataxin precursor (B) or mature frataxin (C) abundance as quantitated by densitometric analysis of the blots in A and normalized

with tubulin levels. Data represent the mean ± 1 S.E.M. from three different independent experiments.

D) 293 Flp-In cell line stably expressing frataxin$^{1\text{-}210}$ was transfected with the indicated siRNA pools. 48 hours after transfection cells were treated with 100 nM Actinomycin D for the indicated times. Cell extracts were analyzed by western blot with anti-frataxin antibody. Pre: precursor; int: intermediate; mat: mature frataxin; mat s.e.: mature frataxin, short exposure. E) The graph represents relative mature frataxin abundance as quantitated by densitometric analysis of the blots in A and normalized with tubulin levels. As expected, levels of mature frataxin are increased upon silencing of RNF126. Data represent the mean ± 1 S.E.M. from four different independent experiments. *P*-values were calculated with Student's *t*-test and were statistically significant (*$P<0.05$) compared to non-targeting siRNA-transfected cells.

Supplemental Figure 5 – Related to Figure 3

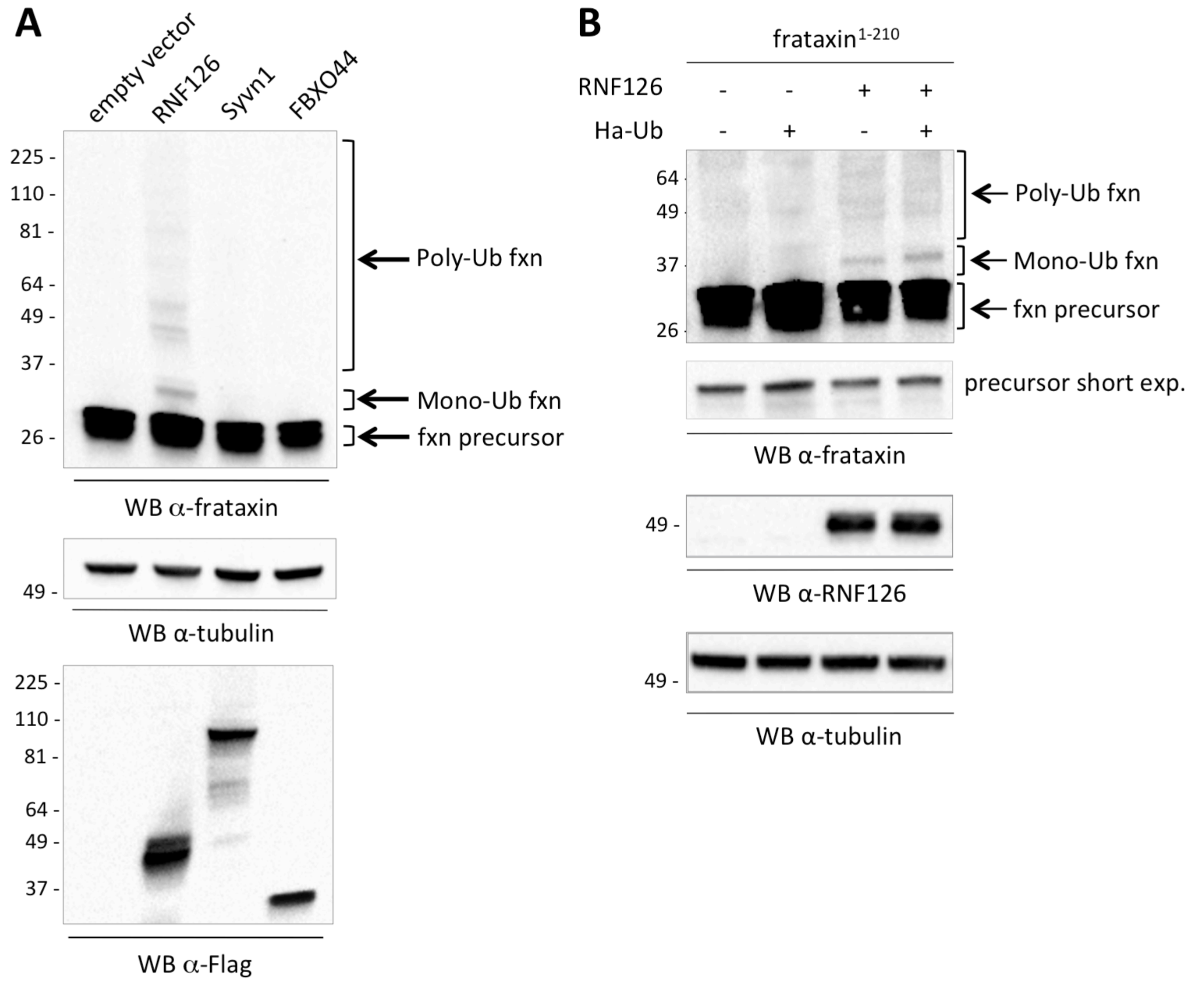

Figure S5. RNF126 promotes frataxin ubiquitination.

A) Expression of RNF126 but not Syvn1 or FBXO44 promotes frataxin ubiquitination. HEK-293 cells were transiently transfected with frataxin$^{1-210}$, HA-tagged ubiquitin (HA-Ub) and either a control empty vector, or the indicated E3 ligase, expressed as Flag-tagged constructs. Protein extracts were collected 40 hrs post-transfection. Total cell extracts were analyzed by WB with anti-frataxin antibody (upper panel), anti-tubulin, as a loading control (middle panel), or anti-Flag to verify the expression of the E3 ligases (lower panel). Slower-migrating bands can be detected above frataxin precursor, corresponding to mono- and polyubiquitinated frataxin.

B) Expression of RNF126 promotes frataxin ubiquitination in the presence or absence of HA-Ubiquitin. HEK-293 cells were transfected with the indicated constructs. Protein extracts were analyzed as in A with anti-frataxin antibody (upper panel), anti-RNF126 (middle panel) and anti-tubulin, as a loading control (lower panel). Slower-migrating bands above frataxin precursor correspond to mono- and polyubiquitinated frataxin.

Supplemental Figure 6 – Related to Figure 6

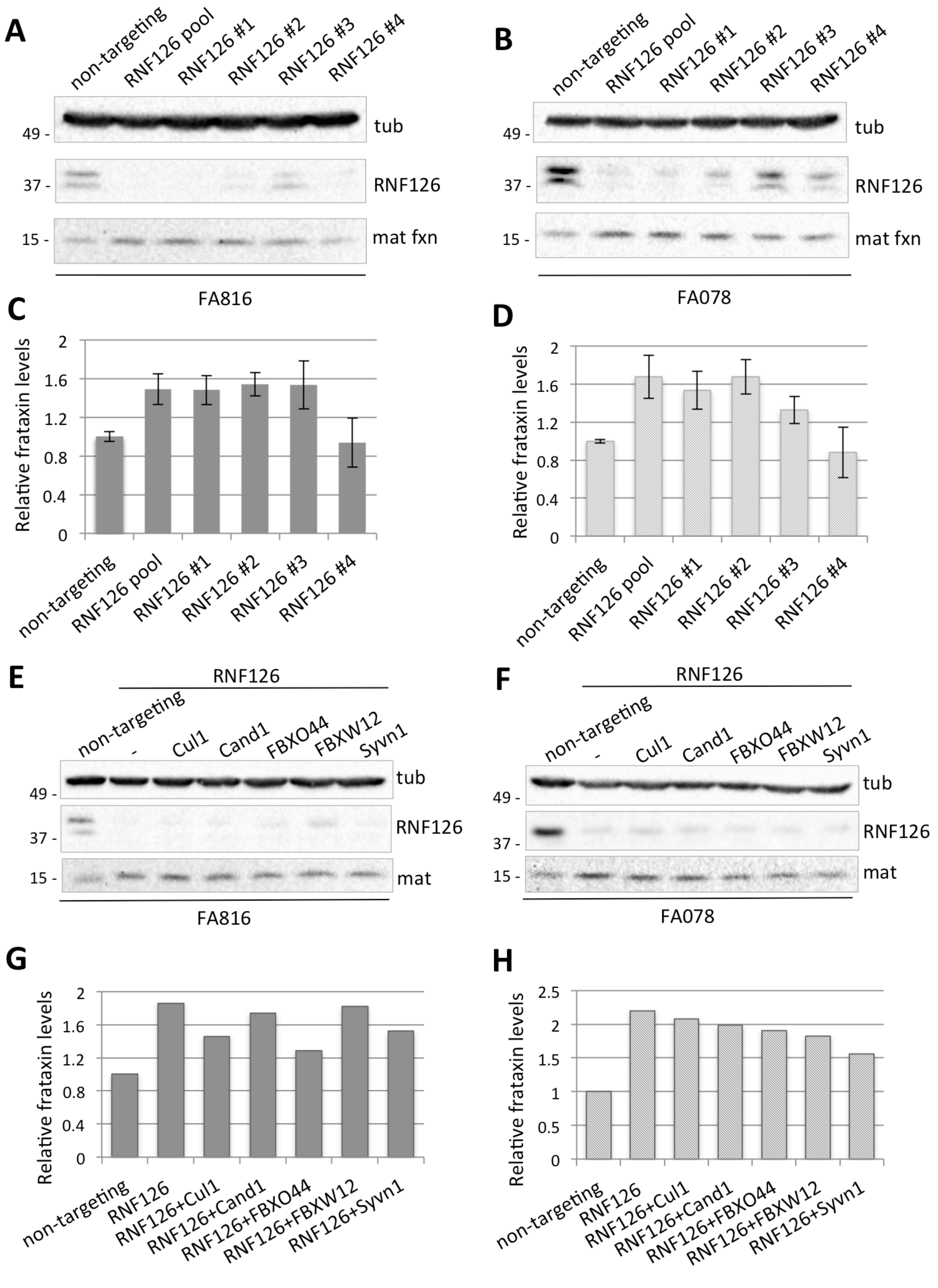

Figure S6. Silencing of RNF126 E3 ligase promotes frataxin accumulation in cells derived from FRDA patients.

A-B) Results obtained in Fig 6 A-B using siRNA pools were confirmed with individual siRNA targeting RNF126. Fibroblasts derived from 2 different FRDA patients, FA816 (A) and FA078 (B) were transfected with 25 nM of the indicated siRNA pool or 25 nM of the individual RNF126 siRNA. Cell extracts were collected 48 hrs post transfection

and analyzed by WB with anti-frataxin antibody, anti-RNF126 and anti-tubulin. Tub: tubulin; mat fxn: mature frataxin.

C-D) The graphs represent relative mature frataxin abundance as quantitated by densitometric analysis of the blots in A and B, respectively and normalized with tubulin levels. Data represent the mean ± 1 S.E.M. from three different independent experiments performed for each patient.

Simultaneous knock-down of RNF126 and the other primary hits does not promote further increase in frataxin levels in cells derived from FRDA patients**:**

E-F) Fibroblasts derived from 2 different FRDA patients, FA816 (E) and FA078 (F) were transfected with the indicated combination of siRNA. Cell extracts were collected 48 hrs post transfection and analyzed by WB with anti-frataxin antibody, anti-RNF126 and anti-tubulin. Tub: tubulin; mat: mature frataxin.

precursor; mat: mature frataxin;

G-H) The graphs represent relative mature frataxin abundance as quantitated by densitometric analysis of the blots in A and B, respectively and normalized with tubulin levels. Data presented are representative of two independent experiments with similar results performed for each patient.

Table S1: siRNA pool target sequences used in this study
Related to Figures 2 and 6, and to Figures S2, S3, S4 (D-E), S6 (E-H).

Gene Name (Catalog Item_Dharmacon)	Target Sequences (5' to 3')	Figure(s)
Non-targeting pool<br>D-001810-10	UGGUUUACAUGUCGACUAA<br>UGGUUUACAUGUUGUGUGA<br>UGGUUUACAUGUUUUCUGA<br>UGGUUUACAUGUUUUCCUA	2, 6, S2, S3, S4 (D-E), S6
CUL1 pool<br>L-004086-00	CAACGAAGAGUUCAGGUUU<br>CGAGGAAGACCGCAAACUA<br>AGACAGUGCUUGAUGUUCA<br>CAUAGAAGACAAAGACGUA	S2, S6 (E-H)
CAND1 pool<br>L-015562-01	GACUUUAGGUUUAUGGCUA<br>CGUGCAACAUGUACAACUA<br>CAACAAGAACCUACAUACA<br>CAUAACAAGCCAUCAUUAA	S2, S6 (E-H)
FBXO44 pool<br>L-019201-00	GAGAGGGCUUCAUCACUGA<br>GCUGAACCCUGACUGGUAA<br>GAAUGGAGGCGAUGAGUGG<br>CAGAUUGCGGGUUCCAAGUA	S2, S6 (E-H)
FBXW12 pool<br>L-032001-00	CAUAUGAGAUCGCAAGUUU<br>GCACAGCCGCAUAACUUUA<br>GGACCAUACUUGUUACUCU<br>GCAAGAGGGUACCAUGAUC	S2, S6 (E-H)
SYVN1 pool<br>L-007090-00	UCAUCAAGGUUCUGCUGUA<br>GAGAAGAGAUGGUGACUGG<br>CAACAUGAACACCCUGUAU<br>GGAAAGGCCUCCAGCUCCU	S2, S6 (E-H)
RNF126 pool<br>L-007015-00	UGUCUAACCUCACCCUCUA<br>CAUCACACAGCUCCUCAAU<br>CGGAUUAUAUCUGUCCAAG<br>GAACAAAACUGCUCCAACA	2, 6, S2, S3, S4 (D-E), S6

Table S2: Individual siRNA target sequences used in this study
Related to Figures 2 and 6 and to Figures S4 (A-C), S6 (A-D).

Gene Name (Catalog Item_Dharmacon)	Target Sequences (5' to 3')	Figure(s)
RNF126 #1<br>J-007015-05	UGUCUAACCUCACCCUCUA	S4 (A-C), S6 (A-D)
RNF126 #2<br>J-007015-06	CAUCACACAGCUCCUCAAU	S4 (A-C), S6 (A-D)
RNF126 #3<br>J-007015-07	CGGAUUAUAUCUGUCCAAG	S4 (A-C), S6 (A-D)
RNF126 #4<br>J-007015-08	GAACAAAACUGCUCCAACA	S4 (A-C), S6 (A-D)
